# Advancing Battery Manufacturing: Synchrotron Characterization for Industry

**DOI:** 10.1021/acs.chemrev.5c00772

**Published:** 2026-02-27

**Authors:** Hyeongjun Koh, James N. Burrow, Nicolò D’Anna, Haozhe Zhang, Tharigopala Vincent Beatriceveena, Jiaqi Wang, Jianwei Lai, Yiming Chen, Jordi Cabana, Maria K. Y. Chan, Ethan J. Crumlin, Paul A. Fenter, Timothy T. Fister, Di-Jia Liu, Ying Shirley Meng, Oleg Shpyrko, Kamila Wiaderek, Kelsey B. Hatzell

**Affiliations:** † Andlinger Center for Energy and the Environment, Princeton University, Princeton, New Jersey 08540, United States; ‡ Energy Storage Research Alliance, 1291 Argonne National Laboratory, Lemont, Illinois 60439, United States; § Pritzker School of Molecular Engineering, 2462 University of Chicago, Chicago, Illinois 60637, United States; ∥ Department of Physics, 8784 University of California San Diego, La Jolla, California 92093, United States; ⊥ Chemical Sciences and Engineering Division, 1291 Argonne National Laboratory, Lemont, Illinois 60439, United States; # Department of Chemistry, 124031 University of Illinois at Chicago, Chicago, Illinois 60607, United States; ∇ 530183 X-Ray Science Division, Argonne National Laboratory, Lemont, Illinois 60439, United States; ○ Chemical Sciences Division, 1666 Lawrence Berkeley National Laboratory, Berkeley, California 94720, United States; ◆ Advanced Light Source, 1666 Lawrence Berkeley National Laboratory, Berkeley, California 94720, United States; ¶ Center for Nanoscale Materials, 1291 Argonne National Laboratory, Lemont, Illinois 60439, United States; ■ Materials Science Division, 1291 Argonne National Laboratory, Lemont, Illinois 60439, United States; ◇ Pritzker School of Molecular Engineering, 2462 University of Chicago, Chicago, Illinois 60637, United States; ● Argonne Collaborative Center for Energy Storage Science (ACCESS), 1291 Argonne National Laboratory, Lemont, Illinois 60439, United States; □ Department of NanoEngineering, 8784 University of California San Diego, La Jolla, California 92093, United States; ▲ Department of Mechanical and Aerospace Engineering, Princeton University, Princeton, New Jersey 08540, United States

## Abstract

Large-scale battery manufacturing requires understanding the fundamental principles of materials and interfaces and relies on advanced techniques for detailed interrogation. Despite advancements in the industrial scale production and their associated quality control tools, challenges such as electrode heterogeneity, internal defects, and large-scale material waste (e.g., scrap) can hamper manufacturing. Synchrotron X-ray characterization techniques offer spatial, temporal, and chemical resolution that can provide diagnostic insights for metrology across various manufacturing steps. This review examines the use of synchrotron tools to advance understanding of key steps in the battery manufacturing process. Recent examples demonstrate how synchrotron methods resolve manufacturing challenges and uncover degradation pathways that are otherwise inaccessible. Future directions for advancing battery manufacturing emphasize collaboration between academia and industry through the use of synchrotron X-ray techniques.

## Graphical abstract

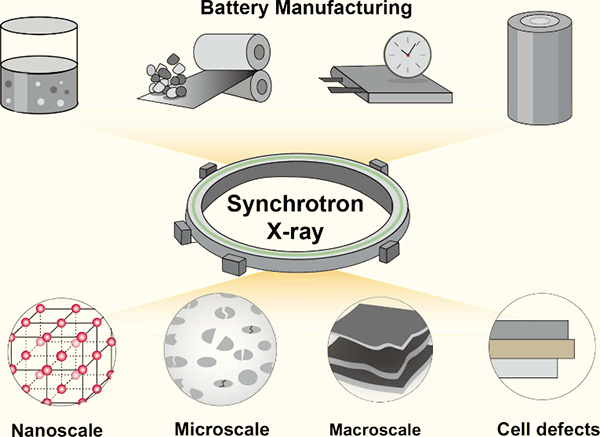

## 1 Introduction

Demand for batteries is rising with growth projected in applications related to back-up power at data centers, passenger vehicles, and aviation. The global energy storage market is expected to grow 7 times by 2035 compared to 2023.^1,2^ While advances in engineering and cell chemistry have significantly lowered prices, the reliability of battery manufacturing remains an underemphasized challenge.^3–5^ A lack of reliability in the manufacturing processes potentially results in (i) safety hazards,^6^ (ii) premature failure,^7^ and (iii) reduced profitability.^4^ Therefore, identifying issues and challenges in industrial processing is critical.

Although quality can be monitored through a combination of inline and offline measurements throughout manufacturing steps,^3,8,9^ advances in synchrotron characterization techniques have made it possible to probe materials and devices at multiple scales.^4,5,10,11^ These methods enable characterization of functional batteries from the macro- to the nanoscale in both the lab- and large-scale cell format. These insights reveal critical insights into cell performance, quality assurance, and safety. Such applications include the identification of internal short-circuits,^12^ assessment of cell failure during thermal runaway,^13^ and structural analysis during various manufacturing steps.^14^

This review highlights how synchrotron characterization techniques can aid in understanding complex processes that are involved in battery manufacturing. From electrode formation to cell-level electrochemistry, advanced X-ray interrogation techniques can provide deeper insights into battery manufacturing. The review offers a comprehensive overview of how these techniques can be applied across the manufacturing process. Although our focus is on industrial Li-ion batteries, these synchrotron approaches are extendable to emerging battery chemistries such as Na-ion and solid-state systems.

## 2 Overview of Battery Manufacturing

Battery production follows a sequence of processes that directly affect the performance of the final product. From an industrial standpoint, the overall process can be divided into four key stages: electrode manufacturing, cell assembly, cell formation, and performance evaluation and validation (Figure 1a).

**Figure 1:**
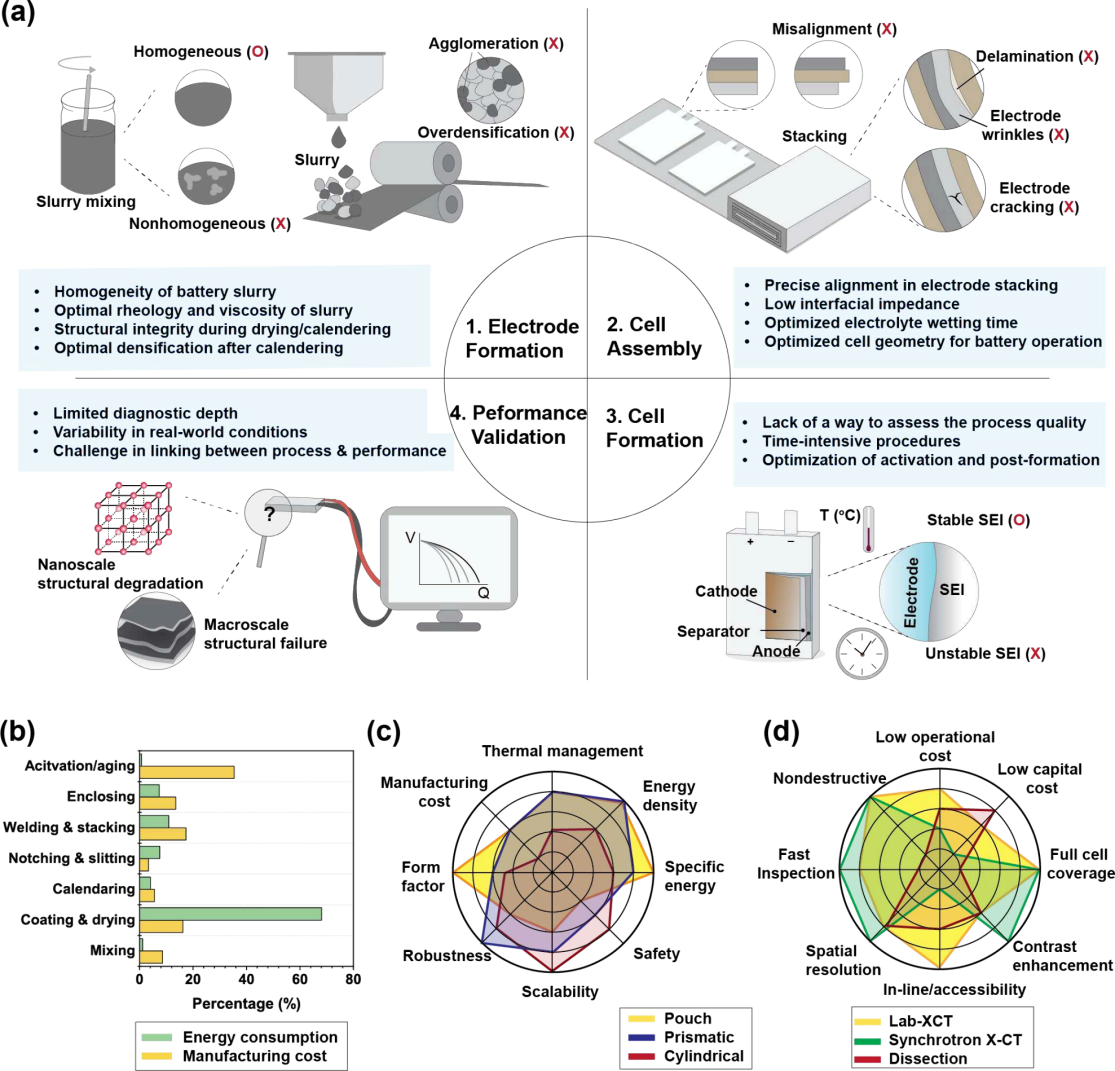
Battery manufacturing process and associated challenges. (a) Key challenges in achieving reliable battery manufacturing. (b) Cost estimates and energy consumption across different stages of the battery manufacturing process, based on calculations from ref 15–18 . (c) Comparison of characteristics among three representative cell designs. (d) Advantages of synchrotron X-ray computed tomography (XCT) compared to other imaging techniques.

### 2.1 Electrode Formation

Conventional electrode processing involves roll-to-roll (R2R) wet-coating processes. Battery electrode materials, binders, and solvents are combined into a colloidal ink and mixed using high-shear mixers to ensure uniformity.^19–23^ The slurry or ink is then cast via slot-die coating onto continuous metal foil and dried using convective hot air and infrared radiation (IR) to evaporate the solvents and form a film. The cost of operating a plant depends on the speed of production. Advanced production lines are pushing lines speeds above 100 m/min.^24,25^ Achieving these speeds without introducing defects such as coating breakup or nonuniformity requires precise control of processing parameters.^26^

Following solvent removal, electrodes are densified in a step known as calendering. Calendering is a compaction process which directly influences the electrode microstructure. Microstructure, specifically porosity and pore size distribution is important for achieving effective ion transport and high power density.^27–29^ However, mechanical stress during compaction can cause fracture of active materials and overdensification.^30,31^ The calendaring process plays a critical role in determining overall electrochemical performance by affecting the ionic and electronic conductivity of the electrodes.^32,33^ Thus, postcalendering characterization is vital for understanding how manufacturing parameters influence electrode performance.

After densification, electrodes are cut into battery-size sheets.^30^ These sheets are then shaped to include slots or notches, depending on the final cell configuration. A range of geometries are considered for industrial applications including prismatic, cylindrical, or pouch cells. The specific geometry or architecture determines the slitting and notching process.

Despite widespread adoption of wet processing in conventional Li-ion batteries (LIB) manufacturing, the drying process is cost-intensive (Figure 1b). Transitioning to solvent-less and solvent-free methods (i.e., dry electrode processing) can reduce manufacturing cost by 6–7%.^34–39^ One strategy to avoid wet processing is to use extrusion processing with high solid content feedstocks or solvent-free electrode formation. Solvent-free methods have been pioneered by Maxwell and Tesla.^40^ While these methods significantly reduce processing time and energy consumption, achieving electrode uniformity remains challenging.^35,41^ Dry electrode processing enables the fabrication of thick electrodes with more uniform microstructure, improving percolation pathways for ion and electron transport.^26,35,41^

### 2.2 Cell Assembly

Currently there are three primary cell formats dominating the industry: cylindrical, prismatic, and pouch cells.^42^ Figure 1c summarizes the key features of these configurations.^43,44^ Among these formats, cylindrical cells are the most widely adopted: the positive and negative electrodes are wound in a spiral configuration and separated by a polymer-based separator. Thanks to decades of manufacturing refinement, cylindrical cells are the most cost-effective to produce, which require less capital cost compared to the other cell formats.^45^ As prismatic cells have high-packing density, this trend may change with high capacity cells. Less hardware costs from cell casings, caps, and seals are needed per capacity (kWh) in prismatic cells.^46^ Their wound structure in the cylindrical format also provides more isotropic mechanical integrity compared with other cell formats. However, a key drawback is the limited heat dissipation, which results in temperature gradients and uneven electrochemical reactions during operation.^44,47^

Prismatic and pouch cells both feature a rectangular form factor, but they differ substantially in internal structure and packaging design. Prismatic cells are enclosed in rigid aluminum cans. This provides the highest mechanical integrity among the three formats. Their stacked electrode design reduces manufacturing steps such as welding, enabling higher energy density.^44^ In contrast, pouch cells use flexible aluminum-laminated plastic films that are more vulnerable to external damage.^48^ Nevertheless, they offer more efficient heat dissipation due to their flat geometry and the lightweight packaging.^49^

Assembly typically involves stacking or winding electrodes alternating with separator layers. Precise alignment is critical to minimize the risk of Li plating, dendrite formation and cell failure caused by electrode area mismatch. Misaligned electrode areas increase the risk of lithium dendrite formation.^12^ Wrinkles or misaligned foils during stacking can compromise the structural and electrochemical integrity of the cell. After stacking, current-collector tabs are welded using ultrasonic or laser welding to ensure low-resistance electrical connections.^50^ Proper weld placement and quality are vital for maintaining performance and minimizing localized heating during operation.

The final step in the cell assembly is sealing. In pouch cells, three edges are typically sealed first, followed by electrolyte filling through the remaining opening. For cylindrical cells, the wound electrode assembly is inserted into a metal can, after which the electrolytes are directly injected. The can is then sealed with a cap and gasket to ensure a tight seal. A major challenge is optimizing wetting so the electrolyte fully penetrates the porous electrodes and separator.^51^ Complete wetting may require several hours to more than a day before formation begins.^17,51,52^ Therefore, estimating quantifiable indicators to estimate wetting time is imperative for efficient manufacturing.

Defect formation during cell assembly remains a critical bottleneck. Even a 2–5% scrap rate in a 38 GWh/year facility translates to millions of faulty cells annually.^4^ Common failure modes, including coating misalignment, particle protrusion, and tab overhang, are more likely to occur at high production speeds. To mitigate yield loss, inline metrology tools are deployed for continuous quality checking monitoring.^3,50^ However, integrating these inspection tools can slow the roll-to-roll process.^4^ Therefore, high-speed and real-time quality control systems that can run at full line-speed operation are needed. Such inline systems ensure that stringent performance and safety standards are met.

Although inspection of upstream components is important, full-cell inspection after assembly is particularly essential. Especially, imaging techniques are pivotal for identifying manufacturing defects after assembly. Various techniques are used, including dissection, X-ray imaging (synchrotron and lab-based),^7^ and acoustic imaging.^50^ Figure 1d summarizes the key attributes of these inspection techniques. Dissection involves physically opening cells to examine internal components, often using electron microscopy. In some cases, cells are embedded in epoxy and sectioned to obtain cross sections. These methods are inherently labor intensive and destructive. In contrast, X-ray imaging evaluates the manufacturing process nondestructively at the full-cell level.^4^ Although it involves high capital costs, this technique is scalable and can be integrated into production lines.

### 2.3 Cell Formation

Cell formation is the process in which fully assembled battery cells are electrochemically activated, and consists of two processes: activation and conditioning (postformation aging). These stages account for a major portion of the overall manufacturing process (approximately 32% in cost while contributing less than 1% of the total energy consumption).^17^ During activation, battery cells are slowly charged to form stable solid-electrolyte interphases (SEI) according to manufacturer-specific protocols.^53^ This process yields inorganic and organic SEI compounds, and gaseous products.^54^ A major challenge in this step lies in assessing the stability and quality of the SEI. Empirical electrochemical characterization methods are required to assess the process. Sub-optimal activation procedures can result in excessively thick SEI, leading to poor electrochemical performance due to hindered ion transport and sluggish kinetics. On the other hand, an overly thin SEI can be mechanically fragile or nonpassivating, resulting in continuous electrolyte consumption leading to electrolyte dry-out.^3,55^ Following activation, gaseous products formed during SEI formation are removed by placing the activated cells under mild vacuum (0.1–0.3 atm). Incomplete removal of these gases can disrupt internal pressure distribution and degrade device integrity and performance. Following activation, conditioning (post-formation aging) involves resting activated cells for a prolonged time to monitor potential manufacturing failures and serves as the final quality-testing step before shipment. Cells are connected to a cycler to measure their open-circuit voltage (OCV).^56^ Cells fail the quality test if the voltage change (mV/time) falls outside the allowed range. Because this process requires extended inspection time, advanced multimodal characterization techniques accelerate the discovery of relationships between processing parameters and resulting device performance, thereby lowering manufacturing costs and boosting throughput.

### 2.4 Performance Evaluation and Manufacturing Feedback

Once formation is complete, battery cells undergo a final stage of performance evaluation and validation, which ensures that only high-quality cells proceed to module and pack assembly. Even if the battery cells pass quality control throughout the electrode and cell manufacturing process, variation in long-term aging trends still exists. Once cells are enclosed in their casings, they become inaccessible, making it difficult to detect performance variations among cells from the same batch. Consequently, parametrization and identification of performance are necessary to evaluate battery reliability.

Performance validation is typically conducted by measuring current and voltage at different C-rates and temperatures.^3^ This step also includes impedance spectroscopy, and thermal profiling. Manufacturers implement automated grading protocols based on key metrics such as capacity retention, internal resistance, self-discharge rate, and open-circuit voltage stability. Such validation is often performed blindly because electrochemical measurements provide only average cell behavior, while local structural and chemical degradation may go undetected. However, such localized effects can be critical to failure mechanisms. Thus, identifying the underlying causes requires advanced characterization, which enables targeted solutions.

From a manufacturing perspective, performance evaluation is not merely a final screening step but a critical feedback mechanism that informs upstream decisions in materials synthesis and electrode design. Variations introduced during co-precipitation, calcination, and slurry mixing can propagate into measurable differences in capacity retention, impedance growth, and rate capability. For example, heterogeneity in precursor particle size or composition may lead to nonuniform grain boundaries,^57^ while deviations in thermal treatment can influence defect density, microcrack formation, and surface reconstruction during battery cycling.^58^ Likewise, cell-assembly factors such as electrode alignment and coating uniformity affect local current distributions.^59^ Because these manufacturing-induced variations often manifest as subtle, spatially localized degradation pathways, traditional electrochemical tests alone are insufficient to diagnose root causes. Thus, linking performance evaluation with materials- and process-level indicators is essential for establishing robust manufacturing and guiding iterative optimization across the production chain.

## 3 Overview of Synchrotron X-ray Characterization Techniques

Synchrotron X-ray techniques offer a unique combination of high spatial and temporal resolution, deep penetration, and the ability to resolve different chemical species. These capabilities distinguish them from conventional analytical methods (Figure 2a). These strengths make synchrotron methods uniquely suited to probe the structural, chemical, and morphological evolution and defects of battery materials in both pristine and processed states, often under *operando* or *in situ* conditions.

**Figure 2:**
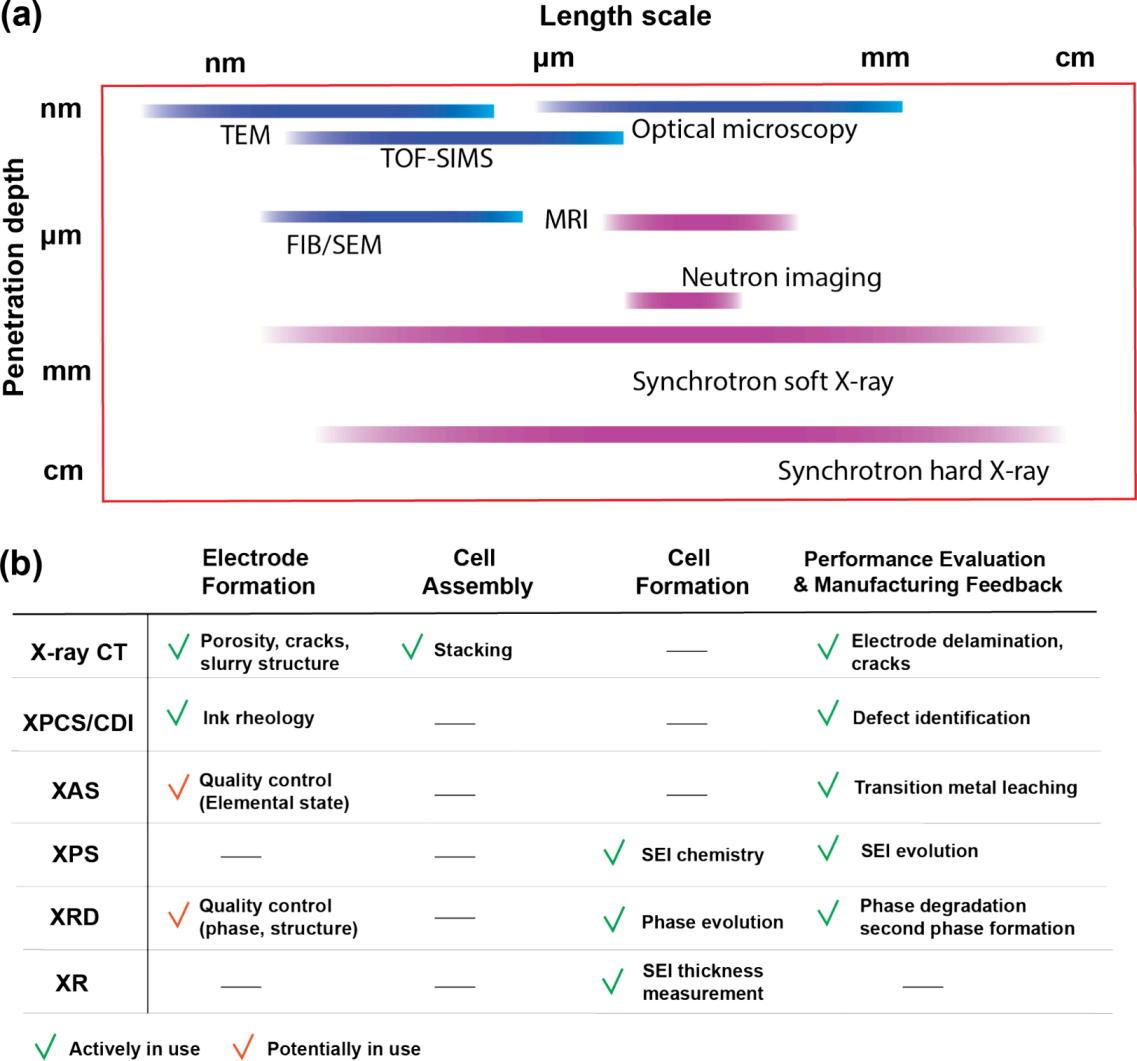
Strengths in synchrotron X-ray characterization and their application in battery manufacturing. (a) Characterization techniques and their corresponding penetration depth and spatial resolution. The purple label indicates potential for nondestructive characterization, while the blue label denotes destructive characterization methods. (b) Synchrotron X-ray characterization in the manufacturing process.

### 3.1 X-ray Diffraction

X-ray diffraction (XRD) probes the crystal structure of matter, enabling analysis of long-range order in atomic-scale structures.^60^ One primary advantage of synchrotron XRD is its ability to detect subtle or transient structural changes that are often missed by laboratory instruments. This high sensitivity makes synchrotron XRD invaluable for capturing fine structural details during material processing and battery operation. It enables detailed characterization of active-material crystal structures, allowing researchers to track structural evolution during cycling and identify key features such as lattice distortions, phase transitions, and strain development. This capability is especially powerful when employed in *operando* and *in situ* modes, which enable real-time monitoring of storage mechanisms, structural degradation, and strain accumulation under realistic conditions.^10^ The high spatial resolution and high flux of synchrotron beams enable depth-resolved studies, making it possible to probe electrode homogeneity across the electrode thickness. The XRD measurements at both beginning-of-life and end-of-life stages in batteries have been routinely conducted in coin cells and single layer pouch cells.^61,62^ Running XRD measurements on cylindrical and prismatic cells requires additional caution, as these geometries can introduce multiple artifacts, including smeared diffraction peaks arising from overlapping signals from different regions.^63^ In addition, steel casings can significantly attenuate X-ray intensity and reduce diffraction signals from internal components.^63^

Synchrotron XRD offers dramatically shorter data-acquisition times than laboratory diffractometers. This enables observation of fast reactions and dynamic processes on relevant time-scales that would otherwise be missed with slower measurements.^64^ Furthermore, the high-flux, high-energy X-rays available at synchrotron facilities enhance penetration through battery components, allowing characterization of a wide range of cell configurations with minimal or no hardware modification.^65–67^ These advantages make synchrotron XRD a uniquely powerful technique for studying batteries across multiple length and time scales.

Synchrotron XRD also enables a range of advanced measurement geometries that expand its applicability in battery research. Microdiffraction with focused hard X-rays (typically at tens of keV) provides micrometer- to submicrometer-scale spatial resolution,^68^ allowing researchers to probe individual secondary particles or localized regions with distinct structural states.^69^ Grazing-incidence XRD (GIXRD) is highly surface-sensitive and can selectively characterize interfacial reactions such as (de)­lithiation processes at the surface.^70^ For systems where components lack long-range order or do not strictly satisfy Bragg conditions, pair-distribution function (PDF) analysis becomes essential.^71^ PDF is particularly powerful for investigating short-range structural motifs and amorphous or nanocrystalline phases that play key roles in battery degradation, such as Li­(NiMn)_0.5_O_2_ and LiMoS_2_.^72,73^

To complement these techniques, Laue diffraction and energy-dispersive XRD (ED-XRD) provide additional flexibility for probing structural states in complex battery environments. Laue diffraction uses a polychromatic beam to simultaneously excite multiple reflections, enabling rapid mapping of crystallographic orientation and strain heterogeneity.^74^ Compared to powder XRD, the variation in X-ray energy beam can capture data collection without rotating specimens. It can be used to identify crystal orientations of metal electrodes and solid electrolytes.^75^ ED-XRD instead varies the incident energy at a fixed scattering angle, allowing fast acquisition of statistically averaged diffraction signals.^76^ Because the outgoing diffracted beam can be precisely defined, ED-XRD can selectively probe regions where thick or encapsulating components are less likely to dominate absorption or scattering. Thus, these methods expand diffraction capabilities to geometries or crystalline systems not readily addressed by conventional powder diffraction. Collectively, these advanced diffraction approaches provide deep insight into dynamic structural phenomena that govern electrochemical performance, such as inhomogeneity tracking in Li­[Ni_1–*x*–*y*_Co_*x*_Mn_*y*_]­O_2_ (NMC) and LiFePO_4_ (LFP).^77,78^ While these advanced techniques have accelerated fundamental understanding of battery materials, translating such insights into manufacturing relevance requires ensuring that experiments are performed under realistic cell configurations and processing conditions.^79^

Each technique also has inherent constraints. Typical synchrotron X-ray beam sizes range from tens of nanometers to submillimeter scales, which are insufficient to directly probe meter-scale electrode rolls used in roll-to-roll manufacturing. For example, a throughput of ∼70 m min^–1^ is typically required during calendering,^80^ whereas only several mm^2^ of electrode area can be only inspected within a similar time frame (a minute) for postpressing quality evaluation. Consequently, XRD must often be complemented by volumetric or imaging techniques such as X-ray tomography to obtain statistically representative information across large areas. Even with rapid scanning, ensuring sufficient sampling to capture electrode heterogeneity remains challenging.^4^ Additionally, several advanced diffraction geometries impose further limitations: grazing-incidence XRD requires smooth and flat surfaces, whereas commercial electrodes exhibit significant roughness and porosity.^81^ Laue diffraction necessitates grains larger than the probe size, restricting its applicability to highly polycrystalline or nanoscale materials.^82^ Finally, many battery components, particularly electrolyte decomposition products, are amorphous or poorly crystalline, making them difficult to detect or quantify by diffraction alone.

For manufacturing applications, measurements should be conducted on thick, calendared electrodes using commercial binder and solvent systems, rather than idealized thin films. Ideally, full-cell stacks or pouch-cell configurations should be employed to accurately represent factory-produced batteries. This requires close collaboration between manufacturing teams and beamline scientists to design compatible sample holders, define cycling protocols, and select beamline configurations that match the material and process scale. Interpreting synchrotron XRD data requires expertise in crystallography and advanced analytical tools such as GSAS-II.^83^ Effective analysis may require techniques including background subtraction, peak fitting, and Rietveld refinement and can be greatly enhanced by simulations or machine learning.^84^ For this reason, manufacturing teams are encouraged to partner with academic institutions or national laboratories with expertise in synchrotron data interpretation.

### 3.2 X-ray Absorption Spectroscopy

X-ray absorption spectroscopy (XAS) is an element-specific technique that provides insights into the oxidation states, local atomic environments, and electronic structures of materials. It works by measuring the absorption of X-rays as a function of energy when X-ray photons interact with specific core-level electrons.^85^ Unlike powder XRD, which probes long-range periodic order, XAS focuses on the short-range structure of both crystalline and amorphous materials surrounding a specific element, making it particularly useful for studying disordered materials, amorphous phases, and buried interphases. XAS is typically divided into two spectral regions: X-ray absorption near-edge structure (XANES), which is sensitive to oxidation state and coordination geometry, and extended X-ray absorption fine structure (EXAFS), which provides quantitative information on bond lengths, coordination numbers, and local disorder.^82^

XAS measurements can be broadly categorized into soft X-ray and hard X-ray regimes based on the incident photon energy. Soft X-ray XAS typically covers energies below 2–3 keV, while hard X-ray XAS is performed at energies above 5 keV. Because of the strong absorption of soft X-rays by air, soft XAS experiments are generally conducted under high or ultrahigh vacuum conditions. In contrast, hard X-ray XAS benefits from significantly greater penetration depth and can be performed under ambient or near-ambient environments, making it more experimentally versatile for *operando* battery studies.^82,86^

Hard X-ray XAS, most commonly conducted at transition-metal K-edges, is widely applied in battery research to monitor bulk redox reactions during electrochemical cycling. Hard XAS measurements can be performed in either transmission or fluorescence detection modes. Transmission geometry follows Beer–Lambert behavior and requires carefully controlled sample thickness and sufficiently high concentrations of the absorbing species.^82,86^ Fluorescence detection, in which the sample is typically oriented at ∼45° relative to the incident beam, is preferred for dilute systems or samples in which most incident photons are absorbed by surrounding components, such as organic electrolytes or thick current collectors in battery cells.

Because hard X-rays probe deep core-level excitations and bulk chemical states, hard XAS is particularly well suited for *operando* investigations of transition-metal redox processes in layered cathodes such as NMC and LFP under industrially relevant cycling conditions.^87,88^ For example, fast *operando* XAS measurements on LiNi_1/3_Mn_1/3_Co_1/3_O_2_ cycled at rates up to 30 °C revealed that the Ni K-edge exhibits pronounced energy shifts, whereas the Co and Mn K-edges remain largely unchanged. This observation suggests that Ni redox dominates high-rate performance and that increasing Ni content can improve rate capability. Time-resolved XAS studies of LFP cathodes have similarly shown that changes in Fe K-edge XANES track lithium content and confirm a two-phase reaction mechanism, in good agreement with diffraction-based observations.^64^ Yu et al. further demonstrated that lithium-ion transport is a key rate-limiting factor governing (de)­lithiation kinetics in these systems.^87^

Probing local structures can be enabled through hard X-ray. An incident X-ray is absorbed in a particular element and photoemission electrons constructively or destructively interfere, creating oscillations above the absorption edge, which is called extended X-ray absorption fine structure (EXAFS). The resulting oscillatory signal can be described using a well-established physical model.^89^ Accordingly, the EXAFS signal can be expressed as a summation over individual scattering paths, where the contribution from each path depends on the coordination number, interatomic distance, disorder, and photoelectron wave vector. By fitting experimental EXAFS data using physically informed structural models, quantitative information on the average local atomic environments can be reliably extracted. A detailed theory of EXAFS can be found here.^89^ A representative example is the study of layered oxide cathodes composed of two crystal structures: Li_1.2_Ni_0.15_Co_0.10_Mn_0.55_O_2_.^88^ This material was found to possess Li_2_MnO_3_ nanodomains, which were proposed to be a rate-limiting factor while experimental evidence had been lacking. The time-resolved EXAFS indicated that Ni^2+^/Ni^4+^ reaction occurred fast within approximately 3 min, while Mn does not significantly participate in the electrochemical reactions. They rather showed very sluggish delithiation kinetics, as evidenced by the slow decrease of the first coordination shell peak.

In contrast, soft XAS probes low-energy electronic transitions, transition-metal L edge (2p→3d) and light-element K-edge such as O, F, and S. The short-attenuation length of soft X-rays provides depths to tens to hundreds of nm. Soft X-ray typically is measured using total electron yield (TEY) or total fluorescence yield (TFY) modes.^10,86^ TEY detects signals from emitted secondary electrons and is highly surface-sensitive. This can render the detection mode suitable for surface reconstruction or CEI/SEI layers. TFY detects emitted X-ray fluorescence photons and provides a relatively deeper probing depth (∼50 nm). This can resolve features within subsurface and near-bulk regions. However, distortions can occur when fluorescence emission from the O K α strongly overlaps with the incoming photon energies.^90^ To reduce this distortion effect, inverse partial fluorescence-yield was introduced, which was found to be effective in resolving Cr, Mn, and Fe L-edges.^91^

A major advantage of XAS for manufacturing-relevant studies is its ability to monitor local chemical processes during high-rate, high-voltage operation. For example, XAS has revealed that Ni^2+^/Ni^4+^ and Mn^3+^/Mn^4+^ are redox-active in NMC cathodes, whereas Co^3+^/Co^4+^ mainly participates at >4.8 V, exceeding the typical electrolyte stability window.^92^ XAS also enables tracking of nonuniform lithiation,^93^ transition-metal dissolution,^94^ and oxygen lattice instabilities^95^ that arise when electrodes are thick, highly loaded, or processed with reduced electrolyte volumes.^96^ These conditions increasingly are adopted in modern manufacturing. These measurements provide direct feedback to mitigate early stage degradation such as surface reconstruction, parasitic reactions, or local redox inhomogeneity.

In addition, XAS can be used to track concentration of doping that may not be detectable by routine energy dispersive spectroscopy (EDS) from scanning electron microscope. Many recently developed cathodes adopt doping techniques with multivalent ions, including Zr, Al, Mg, and Mo to stabilize crystal structures for cycling durability.^97,98^ Dopants are commonly introduced during synthesis through modified coprecipitation steps. For example, transition metal salts (e.g., sulfate-containing precursors: SO_4_^2–^) can be introduced prior to hydroxide precipitation using ammonium hydroxide, where they may act as chelating agents.^99^ In many cases, dopant incorporation occurs after coprecipitation of the transition-metal hydroxides, followed by high-temperature calcination. Typically, dopant concentrations are very small (∼1 at. %),^98^ and this can be below the practical detection limits of EDS. As a result, while widely used for routine compositional checks in manufacturing environments, EDS may lack sufficient sensitivity or confidence to quantify such low dopant levels.

One may use inductively coupled plasma mass spectrometry (ICP-MS) to determine transition metal/dopants ratios. However, ICP-MS does not provide information on whether dopants are structurally incorporated into the cathode lattice or instead remain as secondary phases or unreacted precursor residues. In contrast, XAS can have much higher detection sensitivity (sub ppm level) due to the high flux of X-ray sources,^100^ identifying the minimal concentration of dopants in cathode materials. Furthermore, the oxidation states of dopants determine whether the doping strategy is successful, while XAS combined with complementary techniques such as XRD assesses phase purity and lattice changes. As such, XAS can support manufacturing teams when assessing dopant effectiveness and process consistency in advanced cathode materials.

Despite its growing utility, applying synchrotron XAS directly to manufacturing workflows remains challenging. First, similar to one of the limitations in XRD, typical beam sizes (10–500 μm) are orders of magnitude smaller than meter-scale electrode rolls, limiting statistical representation unless many regions are sampled. Second, cell design modifications are often required due to limited penetration through laboratory-scale materials, particularly for *operando* experiments in sealed pouch-cell formats. As a result, the use of commercial cells for XAS experiments is hindered because of their metal casings. Third, advanced EXAFS fitting requires reliable structural models and extensive computation, demanding expert interpretation. Furthermore, soft XAS requires ultrahigh vacuum condition, which complicates *in situ* or *operando* experiments. Modified cells incorporating SiN window layers have been developed but this still deviates from realistic battery operating conditions.^86^

### 3.3 X-ray Computed Tomography

X-ray computed tomography (XCT) is a non-destructive three-dimensional imaging tool which takes advantage of changes in X-ray attenuation between materials to visualize individual components and materials in a battery.^101^ While focused ion beam/scanning electron microscopy (FIB/SEM) can offer higher resolution (nanoscale), X-rays can provide significant advantages in terms of sample environment. SEM observes samples at surface level so ion beam milling should be combined in order to see through specimens. This leads to significant microscope operation time to polish samples and perform 3D imaging through serial sectioning. FIB/SEM is also a destructive technique. XCT sample volumes can span from less than 500 μm^3^ to a few cm^3^.

In XCT, a series of 2D radiography images are collected at specific angles during sample rotation, and tomographic reconstruction algorithms merge the 2D slices to generate a 3D representation. The contrast of components in battery cells are determined by differences in X-ray attenuation identifying each material phase (active materials, carbon-binder, and pores) in the grayscale image data set.^102,103^ Beyond attenuation-based imaging, several advanced contrast mechanisms can further assist XCT.^104^ Phase-contrast tomography uses the slight phase change in X-ray when passing through the interfaces between materials of similar density. This can reveal faint features such as battery binders and micropores which tend to have weak X-ray absorption contrast.^101^ In addition, diffraction-contrast collects crystallographic diffraction patterns at rotation angles, producing spatially resolved maps of crystalline materials such as cathode materials.

In the battery industry, XCT has become pivotal for quality assurance in various manufacturing processes.^4^ It enables detection of manufacturing defects including misalignment, electrode delamination, and tab misalignment, which are often difficult to identify using conventional dissection methods (Figure 1d). Hard X-rays that can penetrate casings made of aluminum or stainless steel make the technique suitable for non-destructive analysis of industrial cells with form factors such as cylindrical (e.g., 18650) and pouch cells. For example, XCT can be used to monitor electrolyte filling in pouch cells, which is a time-consuming and critical process step.^105^ In addition, XCT can be applied to resolve micrometer features in many manufacturing steps, including slurry mixing, calendaring, particle dispersion, particle distribution, and heterogeneity characterization. While laboratory-based XCT is convenient, synchrotron-based XCT provides superior resolution (down to submicrometer or nanoscale) and faster acquisition due to high photon flux, as highlighted in Figure 1d. For instance, synchrotron-based XCT offers data acquisition times of 25 min while laboratory-based XCT took approximately 250 min even with much larger voxel size of laboratory-based XCT.^106^ In other words, synchrotron-based XCT enables high-throughput characterization of numerous samples and rapid screening of products.

CT has two different length scales. Micro-CT typically operates with voxel sizes ranging from several micrometers down to submicrometer resolution. It uses relatively high X-ray energies and large fields of view, enabling penetration through dense, heterogeneous battery components and allowing volumetric imaging of millimeter-scale samples.^107^ As a result, micro-CT is well suited for statistically representative characterization of statistical analysis of bulk electrode architectures, including porosity gradients, particle packing, and macroscopic manufacturing defects.^108^ Sample volumes accessible by micro-CT can range from hundreds of micrometers cubed to several cubic centimeters, making it compatible with manufactured electrodes and even assembled cells.

This enables visualization of fine structural features similar to what micro-CT does but with high resolution. However, it comes at the cost of reduced penetration depth and a substantially smaller field of view.^110^

While nanoscale X-ray tomography offers significantly higher spatial resolution than conventional micro-CT, this improvement comes with several practical limitations that require careful consideration.^107^ High-resolution nano-CT typically relies on lower X-ray energies and high-numerical-aperture optics, which limit the penetration depth into dense, heterogeneous battery electrodes.^109^ As a result, the technique is generally restricted to imaging thin sections with thickness of often only tens of micrometers. In contrast, micro-CT operates at higher X-ray energies and larger fields of view, allowing real-space characterization of millimeter-scale electrode volumes.^108^ This capability can statistically represent assessment of bulk electrode structures, such as porosity gradients, binder distribution, and calendering-induced deformation across the electrode thickness. For nano-CT characterization of commercial electrodes after calendering and drying, the intact electrode is therefore not fully accessible. Samples often must be milled or microtomed into small pieces to meet thickness and size constraints, and only surface-adjacent or edge regions can be probed effectively. This sample preparation introduces the potential for structural modification, and the restricted field-of-view limits the ability to capture mesoscale heterogeneity. These considerations highlight a central trade-off: nano-CT excels at resolving fine features such as nanoscale cracking and pore architecture, but micro-CT is still essential for volumetric mapping across manufacturing-scale electrodes.

### 3.4 X-ray Photoelectron Spectroscopy

When a sample is irradiated by X-rays of photon energy *hν*, the kinetic energy (*E*_K_) of the emitted photoelectrons is related to the binding energy (*E*_B_) and the spectrometer work function (ϕ) by the energy conservation equation: *hν* = *E*_B_ + *E*_K_ + ϕ. Different characteristic binding energies can be obtained that are both element-specific and chemical-environment-specific, providing quantitative information.

X-ray photoelectron spectroscopy (XPS) is used for battery characterization, particularly in analysis of the SEI and CEI in various battery systems.^111–114^ Due to its intrinsic chemical specificity and surface sensitivity, XPS is ideally suited for probing the electrochemical composition and distribution of interfacial species at the electrode–electrolyte interface.^115^ Conventional XPS is typically performed *ex situ* under ultrahigh vacuum (UHV). However, it has been increasingly upgraded for *in situ* and *operando* studies under ambient-pressure conditions, enabling real-time tracking of interfacial evolution. More details about the principle of ambient-pressure XPS (APXPS) can be found in the literature^116–118^

XPS provides insights into the interfaces that govern SEI and CEI formation, which in turn strongly impact activation processes. XPS is a critical tool for diagnosing favorable interface formation and interface engineering for long-term cycling. In addition, the use of tunable incident photon energy with the high brilliance of synchrotron X-ray sources enhances detection limits and non-destructive depth profiling, enabling depth-resolved analysis.^119^ However, artifacts from argon sputtering should be considered when performing depth-resolved XPS.

The majority of foundational understanding of electrode–electrolyte interphases is derived from UHV-based XPS studies with *ex situ* sample preparation.^111^ However, air-sensitive SEI and CEI formed in battery systems are often unstable in ambient environments with trace amounts of O_2_, CO_2_, and H_2_O.^120^ In addition, trade-offs can exist when washing samples during *ex situ* sample preparation. Washing can enhance SEI/CEI signal intensity,^114,121,122^ but can also result in dissolution of native SEI/CEI species.

Cryogenic XPS (cryo-XPS) has recently been developed to probe preserved SEI composition.^112,123^ By rapidly freezing cycled electrodes, cryo-XPS enables analysis of the native solid–liquid interface without requiring additional physicochemical treatments.^123^ In parallel, synchrotron-based ambient-pressure XPS (APXPS) has been developed to overcome the UHV requirement. The dip-and-pull spectro-electrochemical approach enables *in situ* and *operando* observation of interfacial evolution under near-realistic conditions.^124^ It was introduced for aqueous electrolyte systems^125,126^ and remains still less mature particularly for organic electrolytes as most battery electrolytes are volatile. To date, practical implementation has primarily relied on low-vapor-pressure solvents such as polycarbonate and diglyme-based electrolytes.^127–129^

In addition, the limited spatial resolution at the micrometer scale and radiation damage during ion-sputtering depth profiling remain inherent challenges in the technique.^130,131^ While XPS can provide valuable chemical insights, its direct implementation on commercial cells is constrained by the short escape depth of photoelectrons and the presence of metal casings or thick electrode architectures that block signal detection.^117^ This introduces additional handling steps and the potential for interphase modification.^130^ Therefore, seamless integration of XPS for inline or fully *operando* analysis during electrochemical operation, especially throughout battery conditioning, remains an emerging challenge.

### 3.5 X-ray Reflectivity

X-ray reflectivity (XRR) is analogous of X-ray diffraction and scattering for probing the structure and reactivity of surface and buried interfaces. Unlike Bragg diffraction where the scattering pattern appears as sharp Bragg peaks that reveal the bulk crystallinity of a solid material, the XR signals appear as weak rods of intensity (oriented normal to the surface plane) and reveal direct interface-specific insights into the structure of surfaces, buried interfaces, and thin films.^132–135^ Because of its high interfacial sensitivity and specificity, these reflectivity approaches are particularly valuable for understanding the onset of reactions before reaction products appear as distinct bulk phases. A major challenge of these approaches is the weak reflected signals, necessitating high-brightness facilities (e.g., X-ray synchrotrons).

A typical X-ray reflectivity measurement probes the specularly reflected intensity as a function of incident angle, α. Due to the interference of X-rays that reflect from distinct heights with respect to the surface plane, these measurements are sensitive to the (laterally averaged) density profile across the interface, ρ­(*z*) (where *z* is along the surface normal direction), with vertical resolution of <1 nm. Measurements at small incident angles are essentially described by “slab models”, which are characterized by the electron density, thicknesses of each layer, and their interfacial roughness’s. Here, the interference of X-rays reflecting from the top and bottom of a thin film, for example, leads to oscillations in the reflected intensity with a period that varies inversely with the film thickness.^135^ With increasingly large incident angles, the measurements become sensitive to angstrom-scale structures such as the molecular-scale structure of the film and its relationship to the substrate structure (e.g., epitaxy).

A major strength of XRR is that the long penetration depth of these probes allows measurements to be performed in real time and in the environment of interest, enabling *operando* studies of battery charge/discharge as the system is being cycled. To date, this approach has been used primarily with model battery electrode materials to observe phenomena such as SEI formation,^136^ the initial lithiation of electrodes,^137^ or the onset of conversion reactions.^138^ The application of these tools to the interfaces in solid-state battery systems has also been studied recently.^139–141^ Despite its strong capabilities, XRR is best suited for ideal, flat substrates such as Si wafers. These requirements contrast with practical composite electrodes that contain nanoscale particles, surface roughness, and porous architectures. Ongoing advances in synchrotron optics and geometry design are expected to improve spatial resolution and expand XRR applicability to realistic battery electrodes.

### 3.6 Coherent Diffraction Imaging and X-ray Photon Correlation Spectroscopy

Until recently, most synchrotron X-rays were only partially coherent, and coherent imaging techniques were only available at a few specialized beamlines. With the advent of next-generation synchrotrons, highly coherent X-rays will become ubiquitous, and their use for imaging will become widespread. Coherent diffraction imaging (CDI) and X-ray photon correlation spectroscopy (XPCS) are two techniques that rely on coherence and have been used for *in situ* and *operando* 3D imaging and nanoscale dynamics measurements of crystalline electrode materials.^82^ In addition, XPCS is used to characterize 3D ink printing, as discussed in section 4.4.1. With the current global push toward more intense and coherent synchrotron X-rays, it is expected that both XPCS and CDI will take an increasingly important role in performance evaluation and validation of materials in Li-ion batteries.

Coherent scattering results in a speckle pattern that contains the sample’s structural information. Of particular interest to microcrystalline materials, such as cathode materials, are Bragg diffraction CDI and XPCS, where the speckle pattern is measured around a Bragg peak. In CDI, by rocking the sample in the X-rays, multiple projections of the speckle diffraction pattern are recorded, from which nanoscale real-space images are reconstructed using phase-retrieval algorithms. Specifically, Bragg CDI yields particle shape, electron density, 3D displacement fields, strain,^142^ and defect location maps.^65,143^ Conversely, by recording the temporal evolution of the diffraction pattern, nanoscale dynamics in the sample are measured by X-ray photon correlation spectroscopy (XPCS).^144^ With XPCS, quantitative information is obtained by calculating correlations between diffraction patterns in time, specifically the two-time intensity–intensity correlation function $G\left(Q,t_{1},t_{2}\right)=\frac{I\left(Q,t_{1}\right)\cdot I\left(Q,t_{2}\right)}{{\left\langle I^{2}\right\rangle }_{Q,t}}$ , for a specific detector pixel corresponding to a wave-vector **Q** and where ⟨⟩_*t*_ denotes the average over the total acquisition period. By averaging the calculated *G*(**Q**, *t*_1_, *t*_2_) for all pixels in a **Q** range, |*Q*| dependent two-time correlation *G*(*Q*, *t*_1_, *t*_2_) are obtained.^144^ Modeling of the *G*(*Q*, *t*_1_, *t*_2_) exponential decay yields time scales associated with physical processes, as discussed in section 4.4.1. More in-depth reviews can be found in^145^ for CDI and^144^ for XPCS.

The current understanding of ink formation and the drying process is largely derived from measurements that provide only ensemble-averaged information. XPCS enables time-resolved characterization of rheological properties, providing insights into nonequilibrium dynamics in slurry inks and during solvent evaporation.^146,147^ By detecting nanoscale rearrangements, manufacturers can evaluate the effects of pressure, shear rate, and binder/electrode composition.^148^ In particular, the dynamics of polymer-binder networks and particle dispersion can be quantified through time-resolved XPCS combined with statistical analysis. These insights can guide the rational selection of particle size distribution, binder chemistry, and drying conditions for process optimization beyond conventional trial-and-error approaches. In addition, CDI further allows real-time tracking of manufacturing-induced defects, enabling a deeper understanding of their role in performance degradation.^65,66,143^ This knowledge helps assess when defect minimization is critical and when manufacturing cost may be prioritized over complete defect elimination.

XPCS is a novel experimental technique with some limitations, despite the potential quality-check usage in the mixing step. Reliable XPCS measurements require several minutes to hours depending on the measured system’s time scales,^149^ while in-line inspection during electrode mixing and coating demands throughput on the order of meters per minute in roll-to-roll production. This disparity poses a challenge to integrate XPCS as a routine inspection methodology for quality-check. Consequently, less frequent, beamline-based characterization remains the most practical option at present.

Beyond the fundamental limitations of XPCS,^144^ CDI also faces several technical constraints. Samples are required to contain crystalline particles with submicrometer dimensions, as often found in solid-state electrolytes, and data collection is slow, with typical CDI imaging times in tens of minutes for a single particle.^67^ Additionally, because of the limited penetration depth of X-rays, battery materials often have to be studied in setups that do not always correspond to the final product.^66^ Significant improvements in imaging times are expected with increased photon flux in next-generation synchrotrons, by utilizing machine-learning to automatize imaging procedures (particle alignment and rotation), and with continued improvement of 2D-detector acquisition speeds (currently ∼ MHz). Thus, rather than being tools for continuous quality monitoring, CDI and XPCS are expected to play an important role in providing guidelines for optimization of manufacturing processes.

## 4 Application of Synchrotron Techniques in Battery Manufacturing

### 4.1 Electrode Formation

#### 4.1.1 Ink Formation

Electrode formation encompasses several techniques involving the use of inks. Typically, inks consist of a carrier fluid containing an active material to be deposited on a surface, where the carrier fluid is subsequently removed through evaporation during a curing process.^39^ Ink formulation processes are mainly empirically optimized on measurable macroscale quantities, such as viscosity and surface or interfacial tension. This is because inks are far-from-equilibrium colloidal systems, for which full structural and functional characterization necessitates multiple measurement techniques. In particular, it is necessary to access multiple length and time scales in order to describe interaction mechanisms in inks during and after deposition. Controlled engineering of nano- to mesoscale interactions is required to achieve desired macroscale properties such as performance and durability.^39^ Due to opacity and heterogeneity, characterization methods are limited and include rheology, dynamic light scattering, tensiometry, and cryo-EM.^39^ Recent advances in highly coherent X-ray beamlines and fast detectors,^150^ as well as X-ray diffraction and tomography techniques, are enabling time-resolved characterization of far-from-equilibrium material systems such as inks.

Battery inks are composed of different components, including active-material particles,^151^ conductive carbon,^152,153^ and solvents^154–156^ that can dissolve polymer binders. Consequently, the interactions among these components are important for understanding the rheology of these materials. These interactions can be characterized by the elastic modulus (*G′*) and viscous modulus (*G*″), which describe material response to deformation.^157^ *G′* denotes elastic behavior that stores energy during deformation, while *G*″ represents liquid-like behavior that dissipates energy as flow. Many battery slurries exhibit a transition point at which they change between more liquid-like and solid-like behavior (crossover between *G′* and *G*″) during different processing steps, critically affecting ink formation and casting.^157^ For example, when slurry is cast through a slot-die, high shear rates (on the order of 10^2^ to 10^3^ s^–1^) are applied to the slurry.^158^ Under these conditions, *G*″ is the predominant factor, so that the slurry can be cast smoothly without building up elastic stress that causes undesirable phenomena such as expansion and ribbing of the fluid.^159^ Mild viscoelasticity with sufficient *G′* to maintain structure, together with fast relaxation under high shear rates, ensures uniform and stable coating.

While bulk rheological measurements by conventional rheometers primarily reflect the average behavior of particle ensembles, they provide limited insight into local particle/particle and particle/solvent interactions during ink formulation, mixing, drying, and coating. Consequently, it is beneficial to directly probe how the ink matrix influences local particle dispersity using X-ray imaging techniques. By mapping spatial variations in X-ray attenuation, advanced X-ray imaging can resolve carbon–binder–solvent networks in anode inks as well as active-material and solvent distributions in cathode inks. Such measurements enable quantitative assessment of local particle dispersion, offering mechanistic insights into how microscale heterogeneity governs macroscopic rheological behavior.

Synchrotron XCT provides high-resolution 3D mapping of component distribution and pore morphology, supported by the high photon brilliance of synchrotron sources (Figure 3a). This allows deconvolution of the liquid and solid phases in inks to spatially resolve local heterogeneity in battery slurries, providing unique access to contact-network topology at the micrometer scale. Such information can be obtained by acquiring X-ray radiography images from slurry contained inside thin vessels such as plastic pipet tips (Figure 3b). Consequently, XCT images reveal particle dispersion and agglomeration that directly impact rheological behavior. For instance, at higher mass loadings, when materials become wet granules such as active materials (>70 wt %), the flowability of inks can be significantly hindered,^160^ with large interparticle spacing making the ink behave like a solid, especially at high strain (Figure 3c). Furthermore, XCT imaging has resolved electron percolation networks formed by conductive carbon agglomerates at loadings between 5 and 20 wt % carbon, as shown in Figure 3d,e.^161^ The increase in carbon clustering at higher concentrations reflects the degree of component dispersion, flowability, and ultimately tortuosity, all of which impact downstream processes such as slurry casting and drying.

**Figure 3:**
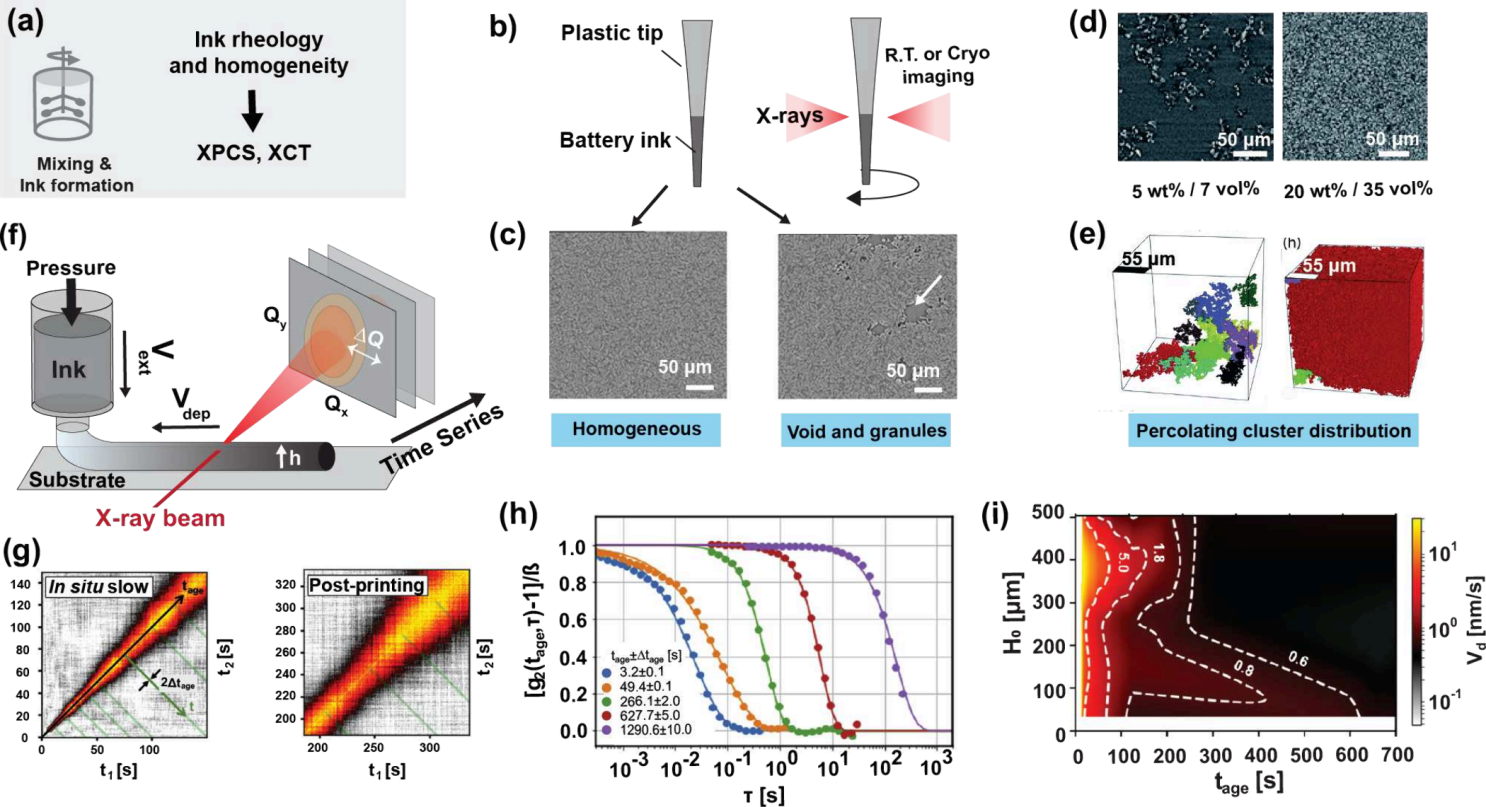
Usage of synchrotron characterization in the mixing and ink formation step. (a) Schematic to describe the mixing step and relevant characterization methods. (b) Steps to perform micro- and nano-XCT on battery inks. (c) Reconstructed slices of battery inks showing distributions of battery composites. Adapted with permission from ref 160. Copyright 2020 Wiley. (d,e) Cross sections (d) acquired from X-ray tomography of electrodes composed of 5 and 10 wt %. (e) Comparison between the two electrodes showing the 10 largest agglomeration reconstructed in the measured volume. Reproduced with permission from ref 161. Copyright 2017 the Royal Society of Chemistry. (f) SAXS measurement setup for inks. Adapted with permission from ref 149. Copyright 2020 Elsevier. An ink under study is extruded onto a substrate at a velocity *V*_ext_ controlled by applied pressure. The substrate moves at a speed *V*_dep_ which controls the deposition speed. Synchrotron X-ray diffraction is measured in transmission on a 2D detector at multiple times. The X-rays incident height *h* is controlled to obtain data at various ink heights. (g) Examples of two-time correlation functions when inks are being dried (*in situ* slow) and after deposition (post-printing), obtained from the setup in (f). Adapted with permission from ref 169. Copyright 2021 Elsevier. (h) One-time correlation *g*_2_ at various times after deposition (*t*_age_) corresponding to green lines in (g), obtained with permission from ref 149. Copyright 2020 Elsevier. The correlation decay time corresponds to a diffusion speed *V*_d_ in the ink, which slows down as *t*_age_ increases. (i) Example of extensive quantitative characterization of diffusion speed *V*_d_ (obtained from fits in (h)) dependence on ink height (*H*_0_) and age *t*_age_. Reproduced with permission from ref 169. Copyright 2021 Elsevier. The results show that curing times are inhomogeneous throughout the ink and are dependent on substrate and atmosphere interactions.

An important factor in micro-CT is the resolution limit in understanding rheology, especially when active materials are small. This becomes more critical as there is growing demand to use nanometer-scale active materials as electrodes.^162^ While synchrotron nano-CT is available to increase spatial resolution, radiolysis of polymers and solvents under the X-ray beam can be problematic.^163,164^ On the other hand, recent advances in cryogenic soft X-ray nanotomography can reach resolutions down to 50 nm, resolving fine features in hydrated and soft materials while minimizing X-ray-beam-induced damage.^165^ The battery inks can be placed in specially manufactured glass capillaries with tip diameters as small as 10 μm, as illustrated in Figure 3b. Capillaries can be plunge-frozen at their tips to vitrify battery inks. As soft X-ray nanotomography requires thin samples, such as several tens of micrometers thick, which alters fluidic behavior due to confinement effects,^166^ an alternative approach to achieve high resolution while avoiding confinement effects is to use cryogenic X-ray photoemission electron microscopy (PEEM).^167^ To complement cryo-PEEM imaging, integration of FIB/SEM can be critical to reconstruct structural details in thick and heterogeneous battery inks.^168^ Although cryo-PEEM and cryogenic soft X-ray nanotomography have not yet been fully leveraged for elucidating nanometer-scale features in the ink, local heterogeneity of battery slurries remains a critical aspect for battery ink formulation.

While XCT can probe battery inks at limited time resolution, two techniques that complement XCT are SAXS (small-angle X-ray scattering) and XPCS, which use coherent X-ray scattering to understand the dynamics of battery inks. For example, SAXS in combination with XPCS was used in lithium titanate (LTO)-based inks for *in situ* and *operando* characterization of out-of-equilibrium processes during 3D battery printing,^169^ where data were collected at varying times and at different heights within the printed ink’s thickness (Figure 3f). Time-dependent SAXS characterizes the evolution of these quantities at the nanometer scale over measurement periods that can extend to hours or days. In the case of LTO-based inks, these quantities are constant in time and independent of ink filament height.^169^

Building on the nanoscale-dynamics information obtained by XPCS, these time- and length-resolved insights are directly translatable to manufacturing control while acquiring SAXS data (Figure 3f). For ink deposition, the one-time correlation function *g*_2_(*t*) is typically described by a single exponential decay of the form *g*_2_(*t*) = 1 + β*e* ^–2(*t*/*τ*_0_)^γ^^, with β a setup-dependent contrast factor, τ_0_ the system’s characteristic relaxation time, and γ the stretching exponent. XPCS analyzes the time-dependent speckle fluctuations in coherent SAXS patterns to extract a characteristic relaxation time (τ_0_) and a stretching exponent (γ), which together quantify how rapidly the ink’s colloidal network forms and relaxes. In the case of out-of-equilibrium dynamics, as is typical in ink deposition, the two-time correlation has a strong time dependence (Figure 3g). For out-of-equilibrium dynamics in ink formation, the *g*_2_(*t*) function calculated around various aging times *t*_age_ yields relaxation times that increase with *t*_age_ (Figure 3h), from milliseconds at deposition to hundreds of seconds after ≈20 min, thus characterizing curing times and highlighting different ink relaxation stages.^146–148,169^

By measuring the evolution of relaxation times and heterogeneity during ink deposition and curing, XPCS allows identification of optimized processing conditions where particle networks form uniformly while minimizing defects such as agglomerates or uneven binder distribution that later lead to electrode cracking or poor adhesion. Additionally, in jammed systems, such as concentrated colloidal inks (gels and emulsions^169^), τ_0_ has a *Q*-dependent (and thus length-scale-dependent) behavior, which is related to a drift velocity. In LTO-based inks, the drift velocity of particles was measured as a function of *t*_age_, ink filament height, and formation direction (deposition or extrusion). It was found to be homogeneous for the first ≈100 s and then to slow down by up to an order of magnitude at different rates near the ink-to-substrate, core, and ink-to-air regions of the filament, with higher speeds near the ink-to-substrate region, as visible in Figure 3i. The results show that the effects of interactions with a substrate or another ink layer are strong and can be characterized with XPCS. Thus, integrating XPCS into electrode production workflows provides real-time, physics-based feedback. Modifying the texture of current collectors is another route to control the behavior of inks on substrates with different surface energies.^170,171^ Adding a thin binder-rich layer prior to ink deposition can also be used to control ink dispersity. Improved understanding of battery inks through these techniques can accelerate optimization of coating, drying, and calendaring steps to deliver more consistent and high-quality battery electrodes at scale.

#### 4.1.2 Drying and Calendaring

Dynamics in slurry drying and calendaring processes can be studied by XCT, providing insights into process quality, as shown in Figure 4a. Typically, the workflow involving XCT in this process uses radiography images that can be further processed through segmentation (Figure 4b).^173^ This workflow enables identification of the spatial distribution of active materials. While homogeneity in particle distribution is mainly established during the drying process, XCT imaging after drying and calendaring enables determination of electrode microstructures, including porosity and tortuosity, and the connections between primary and secondary active materials. This characterization is becoming increasingly important with the rise of dry-electrode processing (section 2.1).^34^ In the absence of solvent-mediated redistribution, the uniform packing of active materials, conductive additives, and binders must instead be controlled through mechanical processing. As a result, detailed 3D structural analysis is essential to ensure that density and porosity are uniformly established across the electrode thickness.

**Figure 4:**
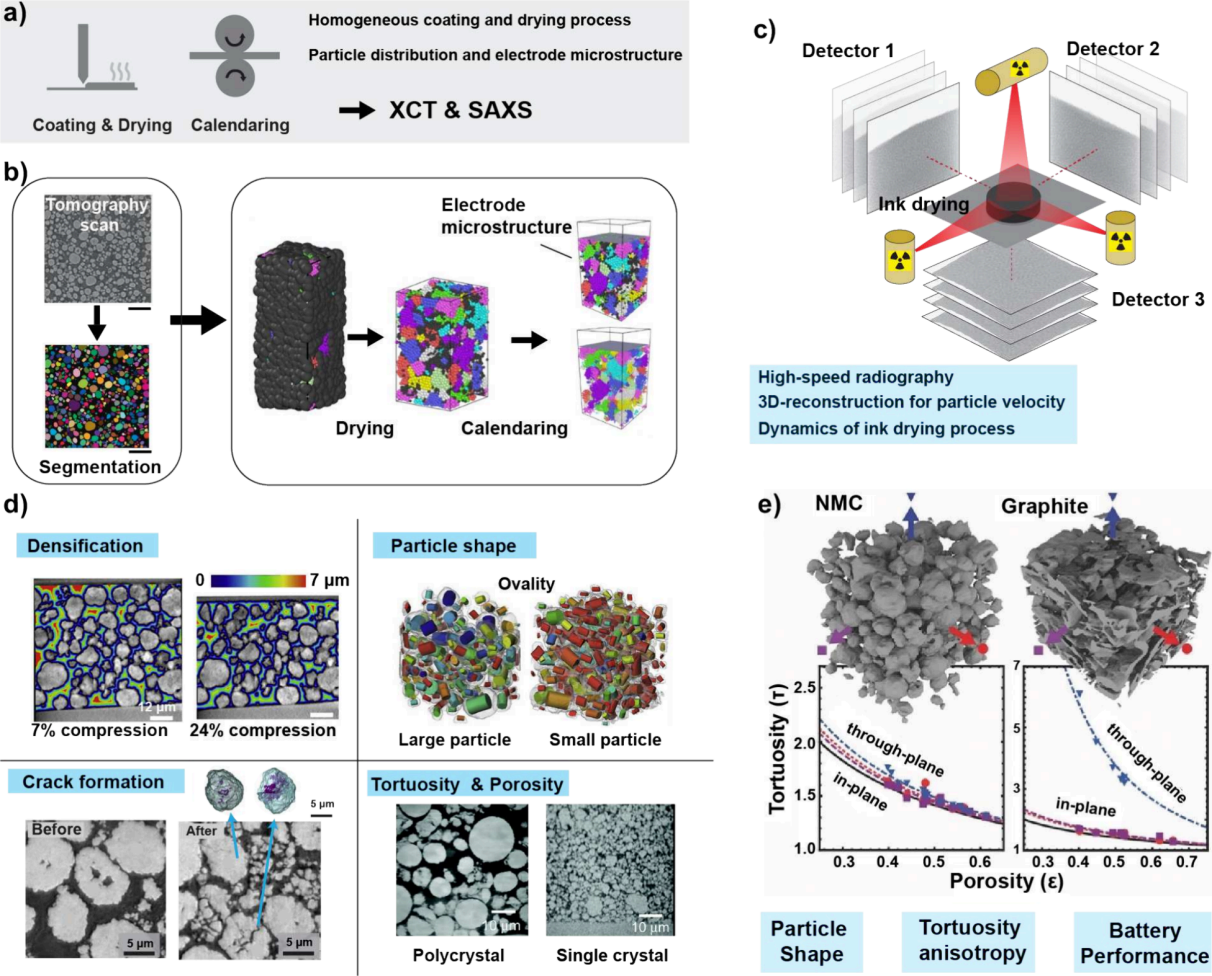
Synchrotron characterization for coating/drying and calendaring steps. (a) Schematic of the manufacturing steps and relevant synchrotron characterization methods. (b) XCT workflows in understanding of ink drying and electrode calendaring process. The right panel in (b) was adapted with permission from ref 172. Copyright 2023 Elsevier. The left panel in (b) was reproduced with permission from ref 173. Copyright 2023 The Authors, Batteries and Supercaps, Wiley-VCH, under the terms of the Creative Commons Attribution (CC BY) License. Schematic diagram of X-ray rheography captured by three orthogonal detectors to improve the spatial and temporal resolution beyond that of conventional XCT. (c) Adapted with permission from ref 174. Copyright 2018 licensed under CC BY 4.0. (d) Insights into the effects of calendering, revealed through XCT imaging. Adapted with permission from ref 173,175 . Copyright 2020 Cell Press and Copyright 2023 Wiley, respectively. Bottom right panel in (d) was reproduced from ref 176. Copyright 2021 The Authors, Royal Society of Chemistry under the terms of the Creative Commons Attribution (CC BY) License. (e) Tortuosity change as a function of porosity between spherical NMC and planar graphite anodes, highlight the significant anisotropy in tortuosity from the graphite electrode. Adapted with permission from ref 177. Copyright 2014 Wiley.

Time-resolved 3D radiographic imaging further reveals how process parameters impact electrode film quality.^160,178–180^ For example, higher solid concentrations result in granules with internal voids that lead to a less homogeneous particle distribution, ultimately reducing the quality of the dried film.^160^ In addition, a lithium-ion slurry composed of a mixture of silicon oxide electrode and carboxymethyl cellulose (CMC) in aqueous dispersion was investigated by XCT.^179^ Radiographic analysis demonstrated nonuniform evolution of electrode distribution under drying conditions. As a result, this highlights that drying alone, without any instrumentation, may lead to poor homogeneity in particle distribution. In the production workflow, applied heat such as infrared (IR) radiation is used in the drying process,^3^ which promotes homogeneous slurry distributions when forming electrodes, although further optimization is required in combining heating rates and vacuum conditions.

However, it requires sample reorientation and long acquisition times, restricting dynamic studies.^174^ Even with short exposure periods and fast time scales, once particles move from their initial positions, CT scans lose accuracy to some extent. To address these limitations, X-ray rheography has been developed to construct 3D reconstructions by combining 2D motion data from radiographs taken at three orthogonal axes, which can reveal displacement fields in viscous fluids (Figure 4c).^174^ This methodology overcomes the slow-scan nature of CT and enables real-time dynamics and deformation mapping of viscous flows in granular media during drying.

While XCT captures the dynamics of battery ink dispersion in liquid suspension, both *in situ* and *ex situ* XCT can offer structural information postdrying and calendaring.^173,175,176,180–184^ Figure 4d demonstrates key structural information obtained by XCT imaging, including densification, particle-shape changes, crack formation, and tortuosity and porosity, which play significant roles in electrochemical behavior in battery cells. At the macroscale, the ion-percolation networks can be described using tortuosity (τ) and electrode porosity (ϵ) in the Bruggeman relation.^185^

$$\tau =\varepsilon^{1-\alpha }$$,

where α denotes a geometric constant representing electrode microstructures. Typically, well-manufactured battery electrodes tend to have α values between 1 and 2, indicating uniform and isotropic movement of ions and liquid electrolytes.^186,187^

Beyond its influence on ionic transport, calendaring directly impacts volumetric energy density by increasing electrode density and reducing pore volume. While moderate calendaring improves particle–particle contact and electronic conductivity, excessive densification can hinder electrolyte infiltration and ionic transport, results in trade-offs between rate capability and energy density. As a result, quantifying electrode porosity and tortuosity is critical for optimizing densification without compromising the trade-off relationship.

Quantifying these structural parameters is especially important for fast-charging batteries, where ionic-transport limitations dominate. For instance, variations in particle-size distribution after calendaring influence deformation behavior.^175^ Narrower particle-size distributions are more tolerant of densification and are preferable for thick electrodes operating at high areal loadings. In contrast, broader size distributions lead to uneven particle rearrangement and mechanical instability. Additionally, excessive calendaring can lead to crack formation, which has a detrimental effect on electrode rigidity. For example, 2D sliced images of NMC electrodes obtained through nano-XCT show obvious cracks after calendaring at 150 MPa, while particles remain intact at 80 MPa.^173^ Particle morphology further affects porosity distribution during calendaring, with XCT-based statistical analyses linking high tortuosity in graphite-based electrodes to their in-plane anisotropic structure, as demonstrated in Figure 4e.^177^ Graphite electrodes exhibit high tortuosity in the through-thickness direction, which is a limiting factor for high-power operation.

While indirect techniques such as mercury intrusion porosimetry have been used to estimate tortuosity,^182^ XCT now enables direct 3D visualization of microstructure–property relationships. Synchrotron-based XCT achieves sub-50 nm resolution but still faces challenges: (i) insufficient resolution for small features, (ii) limited field of view, and (iii) poor contrast between carbon and binder phases.^183,184^ To mitigate these issues, focused ion beam scanning electron microscopy (FIB/SEM) offers higher contrast and resolution for fine features, complementing XCT’s larger statistical field of view. Alternatively, physics-based models can estimate the influence of carbon and binder phases on porosity and tortuosity, reducing reliance on FIB/SEM.^183^

Across the manufacturing steps from ink formation to drying and calendaring, a unifying challenge is mesoscale heterogeneity that can arise during each step. XPCS can detect nanometer- to micrometer-scale variations in particle dispersion during ink formation. XCT visualizes these variations during drying. Postcalendaring tomography can link the heterogeneity to crack formation, particle deformation, and tortuosity. Electrode heterogenity can be related to ink formulation and processing steps. However, no single synchrotron technique can capture this entire evolution alone. Instead, understanding how heterogeneity propagates requires coordinated multiscale measurements that explicitly account for the processing conditions in preceding steps. This underscores that controlling nanoscale colloidal behavior is as critical as optimizing mechanical operations such as calendaring.

### 4.2 Cell Assembly: Manufacturing Architectures and Defects

Architectural heterogeneity remains one of the primary sources of performance variation and early degradation in lithium-ion battery manufacturing. Recent developments in XCT, especially using synchrotron sources, have enabled the detection of structural defects caused by fabrication and stacking with increasing spatial and chemical resolution (Figure 5a). These imaging tools, applied across a range of length scales, have shown how internal geometry influences the distribution of strain, electrolyte, and current within the cell, and how these variations contribute to the initiation and growth of defects. Attia et al., in a recent perspective, argued that CT should be integrated into routine quality control to ensure reliability at gigawatt-hour production volumes.^4^ They noted that the combination of high throughput and sensitivity to minor misalignments makes CT one of the few tools suitable for catching early stage faults before cells are deployed.

**Figure 5:**
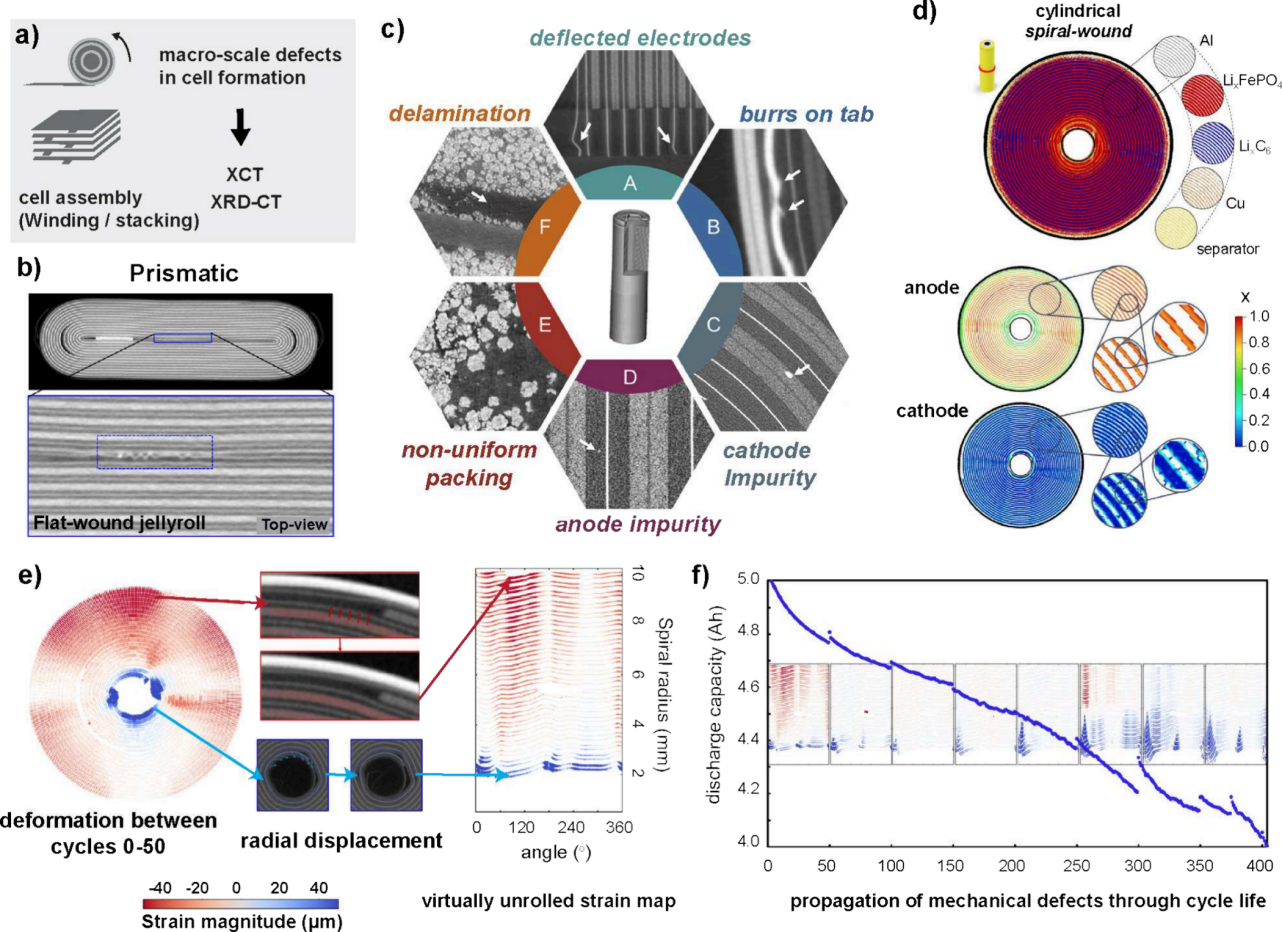
Usage of synchrotron characterization for cell formation. (a) Schematic illustration of the manufacturing process including winding and stacking, and along with associated synchrotron characterization techniques. (b–f) Typical (b) form factors and (c) manufacturing defects prevalent in commercial battery cells as quantified by X-ray computed tomography with either (e) absorption or (d) diffraction contrast and (f) relation to capacity fade and performance degradation. (b) Adapted with permission from ref 7. Copyright 2022 The Electrochemical Society. (c) Adapted with permission from ref 188. Copyright 2021 Cell Press, licensed under CC BY 4.0. (d) Adapted with permission from ref 189. Copyright 2021 Elsevier. (e,f) Reproduced with permission from ref 190. Copyright 2023 The Electrochemical Society, licensed under CC BY 4.0.

At the production level, Kong et al. applied CT to evaluate the pouch-cell quality, showing that small variations in electrode placement, alignment, and coating uniformity caused early stage capacity fade (Figure 5b).^7^ For example, cells with tab misalignment exhibited higher impedance and thermal instability during cycling. At the electrode level, Qian et al. employed a multiscale X-ray CT approach to reconstruct and simulate the 3D morphology of commercial NMC electrodes, uncovering how heterogeneities in porosity and tortuosity drive spatial disparities in (de)­lithiation at elevated C-rates.^191^ Under fast discharge, lithium-ion flux was shown to concentrate in narrow pores and near the separator, leaving deeper electrode regions underutilized and structurally stressed. Simulations based on CT-derived architectures revealed that these transport limitations create local overpotentials and uneven state-of-lithiation, increasing the likelihood of particle cracking and delamination.

Electrode misalignment and internal warping, long suspected to affect local reaction uniformity, have been directly visualized in commercial full cells. In a correlative study combining high-throughput CT with neutron imaging, Ziesche et al. found that electrode layers exhibit measurable shifts in spacing and planar alignment, which revealed internal voids and areas of incomplete wetting.^192^ This work pioneered the use of the “virtual unrolling” technique on a Li/MnO_2_ CR2 primary battery from Duracell to understand how stacking and winding impact the architecture and manufacturing defects of commercial batteries. These geometric inconsistencies were linked to uneven lithium intercalation and early depletion of electrolyte.

Structural defects also arise from impurities and internal inclusions introduced during fabrication. Qian et al. used synchrotron CT and complementary spectroscopy to analyze 18650 cells and identified various key defects prevalent in commercial battery manufacturing (Figure 5c).^188^ Such defects include misaligned electrodes, delamination layers, and burrs on tabs from imperfect welding processes. Additionally, metallic particles that become embedded during electrode calendaring, often as a result of machine contact, can serve as initiation sites for electrode delamination and gas evolution. These inclusions, though microscale in size, created local inhomogeneities in stress and reaction rate that eventually disrupted large areas of the electrode. As battery production scales, detecting and mitigating such defects during the manufacturing process will become crucial for controlling variability that directly impacts cycle life, safety, and downstream reliability.

In addition to quality control via detection of morphological defects, hyperspectral analyses can yield further chemical and structural insight during cell formation and early operation of commercial batteries. For example, Petz et al. used multiscale XRD-CT to study lithium redistribution in 18650 cells (Figure 5d). This study revealed off-center winding and coating nonuniformity that led to asymmetric lithium transfer during high-rate discharge.^189^ The resulting imbalance in lithium availability produced measurable spatial gradients in state of charge, detectable through characteristic changes in the lattice parameters of cathode (LFP) and anode (graphite) active materials. This work highlighted a failure pathway that begins with only minor geometric irregularities in commercial battery production. A further multiscale CT analysis of commercial 18650 cells, conducted by Zan et al., used phase-contrast tomography, nanospectrotomography, and conventional micro-CT to trace defects from the full cell down to the particle level.^193^ This work found that particle cracking, especially in the cathode, often precedes larger-scale electrode delamination and deformation.

Other studies have expanded the scope of CT analysis beyond damage identification to include defect tracking across the full lifecycle of commercial cells. In a 4D CT and digital disassembly study of a 21700-format battery, Kok et al. followed structural changes from formation to end of life, identifying progressive distortion of the jelly roll driven by anisotropic swelling and shrinkage (Figure 5e,f).^190^ This evolution in geometry, though gradual, leads to measurable shifts in internal resistance, underscoring the cumulative effects of mechanical drift. Another defect commonly observed with CT is gap formation between the jelly roll and the cell housing. In a grayscale CT study of 18650 cells, Spielbauer et al. showed that these gaps widen with both state of charge and age, particularly under high-voltage storage.^14^ The loss of mechanical contact between the electrode stack and the housing was attributed to gas evolution and swelling during use. These effects are especially visible in cylindrical cells but likely occur in pouch cells as well, where flexible packaging may conceal similar internal movement.

While pouch cells allow for more uniform current paths, mechanical contact, and thermal management, they still show architecture-dependent aging behaviors. In a study on single-crystal NMC811/graphite pouch cells, Eldesoky et al. used synchrotron CT to examine electrode structure after long-term cycling across a range of depths of discharge and C-rates.^194^ Even under aggressive cycling, there was no visible microcracking or active material loss. This was attributed to both the mechanical strength of the single-crystal particles and the structural flexibility of the pouch format. In contrast to the deformation seen in cylindrical cells, the flat architecture of pouch cells appeared to reduce mechanical mismatch and suppress damage accumulation. Such deformation in jelly rolls can be severe when combined with silicon-containing anodes that accompanies large volume expansion.^195^

In many of these studies, CT was the only technique capable of detecting early stage structural changes. Voltage curves and impedance measurements often failed to capture underlying mechanical or morphological damage, especially when defects were spatially isolated. Across multiple literature, internal defects such as delamination and void formation, were shown to persist unnoticed until they triggered secondary effects such as lithium plating, strain localization, or thermal gradients. When combined with phase contrast or diffraction contrast, CT enables the detection of these subtle features *in situ* or over extended cycles. Recent advances in analysis methodologies, such as radial segmentation and virtual unrolling, expand the ability of CT to link battery geometric features to failure modes. Consequentially, these findings underscore that defects in battery architecture are linked to unwanted electrochemical processes at later stages. Even small structural defects can propagate across different length scales and accelerate the degradation during battery operation.

### 4.3 Cell Formation (Conditioning and Activation)

The activation and conditioning of lithium-ion batteries (LIBs) are critical processes influencing long-term performance, safety, and reliability, especially in high-power operation such as electric-vehicle (EV) applications. Activation and conditioning is the initial electrochemical step that establishes SEI, a crucial passivation layer on the anode and CEI on the cathode, formed through controlled electrolyte decomposition.^114,199^ This interphase plays a critical role in regulating Li-ion transport, minimizing continuous side reactions, and preserving cell integrity over extended cycling.

After the activation process, the conditioning phase in industrial settings allows additional stabilization and chemical maturation of the SEI. During this period, loosely bound species reorganize or dissolve, and more stable SEI components redeposit, leading to a denser and more protective interphase. This process not only enhances cell consistency and safety but also enables the identification and elimination of defective cells before they are used.^54,114,199,200^ In addition to interphase stabilization, the activation process can also facilitate structural relaxation of active materials, particularly cathodes, which often undergo structural reconstruction during initial cycling. This stage provides an opportunity to investigate strategies, such as doping to mitigate degradation and reinforce structural integrity. Although characterization techniques such as EIS, lab-based XPS/XRD, and electron microscopy are commonly used to probe SEIs and structural reconstruction in active materials, synchrotron-based X-ray techniques offer significantly sensitivity, providing deeper insights into SEI chemistry and active-material evolution to qualify the manufacturing process (Figure 6a).^96,196,200^

**Figure 6:**
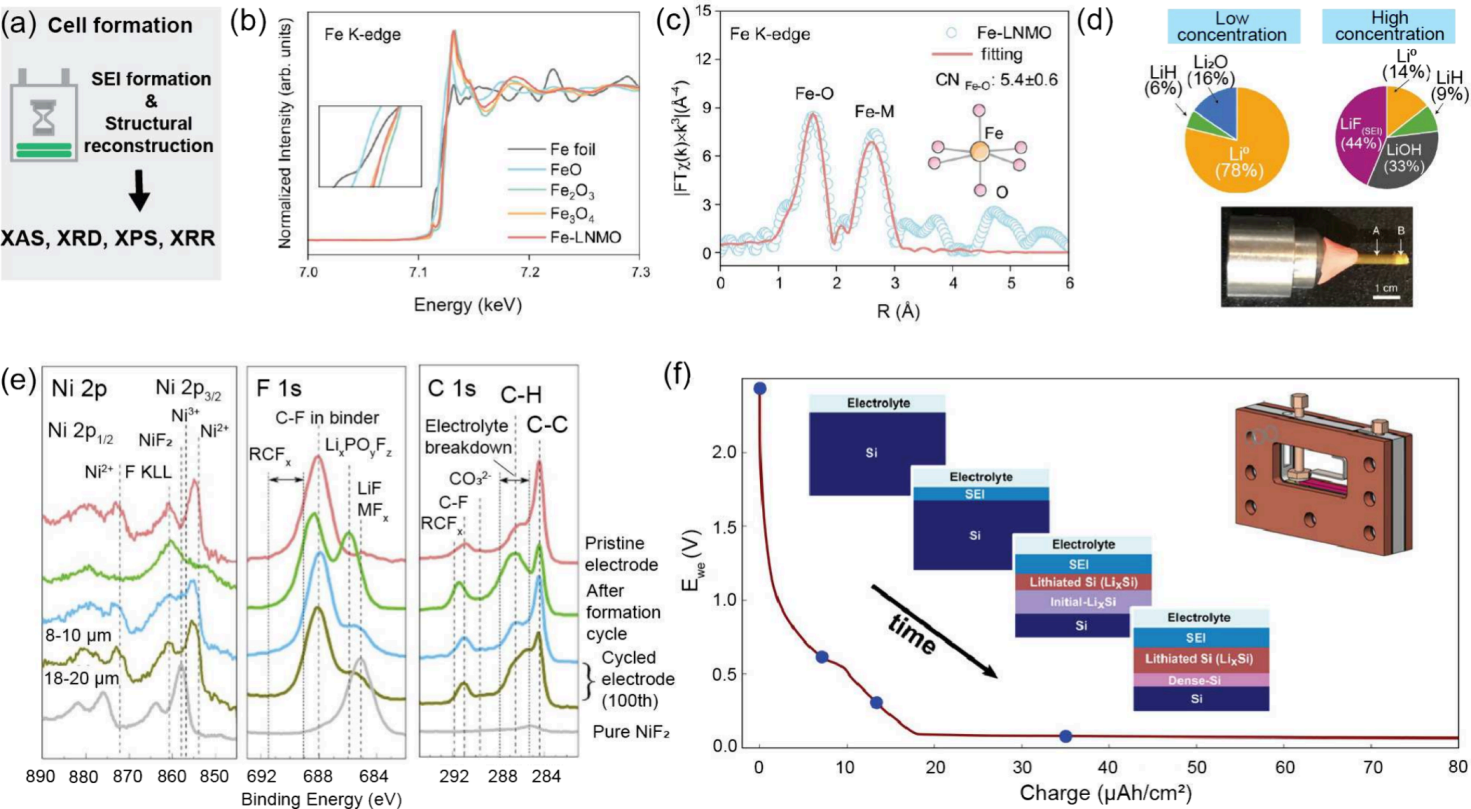
Synchrotron characterization in the conditioning and activation process. (a) Schematic indicating the activation process where the formation of SEI and reconstruction of active materials are important. (b) Fe K-edge XANES of Fe-LNMO in FHFP electrolyte after 100 cycles at 29.4 mA/g between 3.5–4.9 V at 25 °C. (c) Fourier transform magnitude of Fe K-edge EXAFS spectrum of Fe-LNMO (inset represents the Fe–O coordination). (b,c) Reproduced with permission from ref 96. Copyright 2025 Nature licensed under CC BY 4.0. (d) Quantification of SEI components obtained by fitting the XRD. The picture below shows the capillary setup to conduct the synchrotron experiments. Adapted with permission from ref 196. Copyright 2021 Spring Nature. (e) SEI formation and activation process of LiNi_0.7_Co_0.15_Mn_0.15_O_2_. Reproduced with permission from ref 197. Copyright 2017 Nature, licensed under CC BY 4.0. (f) Setup for XRR measurement and its schematic reactions on Si (100) electrode, induced during the activation process Adapted with permission from ref 196. Copyright 2021 American Chemical Society.

For example, XAS and XRD were used to investigate the doping effect on cobalt-free spinel LiNi_0.5_Mn_1.5_O_4_ (LNMO) cathodes paired with lithium–metal anodes (Figure 6b,c). Although LNMO offers a high discharge voltage, its practical application is hindered by issues such as manganese dissolution and excessive electrolyte decomposition at high voltages. To reduce the use of costly elements such as Co in conventional active materials, the development of cobalt-free electrodes is highly desirable, although the aforementioned problems impede this progress. To address these challenges, ferrocene hexafluorophosphate (FHFP) was introduced as a multifunctional electrolyte additive. This additive enables dynamic Fe^3+^ doping into the LNMO lattice during electrochemical cycling, mitigating Mn dissolution and reinforcing structural stability. Fe K-edge XANES and EXAFS were employed to analyze the dynamic Fe doping and its role in stabilizing the electrode–electrolyte interphases. XANES analysis indicated that Fe in the doped LNMO exhibits a mixed valence state (Fe^2+^/Fe^3+^), suggesting partial reduction during cycling (Figure 6b). EXAFS spectra confirmed Fe–O and significant Fe–M (Mn, Ni, Fe) interactions, evidencing lattice incorporation (Figure 6c).^96^ This study provided key structural insights into how the additive impacts CEI formation and the structural properties of active materials during the activation process. The economic feasibility of additives is particularly important for industrial applications. FHFP is derived from ferrocene with iron chloride and ammonium hexafluorophosphate, all of which are commercially available.^201^ Many reported dopants/additives with excellent laboratory performance remain impractical for manufacturing due to limited supply or high cost.^202^ Multiple characterization techniques are necessary to understand the roles additives play on interphases.^50^

Synchrotron XRD enables the identification of nanostructures in the SEI after the conditioning process. Shadike et al. showed the structural composition of the SEI in lithium–metal batteries by utilizing structural modeling and Rietveld refinement of XRD patterns.^196^ They identified the presence of lithium hydride (LiH) and nanocrystalline lithium fluoride (LiF), which had long been under debate. In localized high concentration electrolytes (LHCEs), the SEI primarily consisted of LiH and Li_2_O, whereas high-concentration electrolytes (HCEs) showed significantly higher amounts of nanocrystalline LiF (∼3 nm). Figure 6d depicts the quantification of (nano)­crystalline components in the SEI obtained by fitting the XRD patterns.^196^ The origin of the hydrogen sources in LiH has been attributed to hydrogen gas evolved by solvent decomposition.^203,204^ Industrial battery electrolytes are usually carbonate-based, that can potentially lead to hydrogen gas evolution from SEI formation.^202^ Furthermore, LiH is not electrochemically active once it forms, so its presence causes capacity decay. Therefore, it is important to design new electrolytes and additives that suppress hydrogen evolution during industrial cell formation, beyond what is achievable with conventional carbonate-based electrolytes for Li-metal batteries.

Importantly, a key mechanistic insight in the HCE systems is that Li-ion is transported with the ions surrounded by the several units of anions, leading to anion-derived SEI products.^205,206^ Thus, it leads to SEI products from salt decomposition, rather than from solvent reduction, governing SEI chemistry in HCEs.^196^ Consequently, this provided critical insight into the nanoscale and amorphous structure of SEI components, understanding their structural properties, which directly impact ion transport and mechanical stiffness in the interphases. Despite their benefits, HCEs are challenged by its higher costs and its increased viscosity, limiting its industry adoption. Localized high concentration electrolytes (LHCE) are another routes to resolve these issues.^207^ In LHCE systems, only a fraction of solvents dissolve salts while others dilute viscosity and improve wettability, maintaining the properties of HCE.^208,209^ This can reduce the cost of the overall usage of salt and overcome viscosity issues that limit the ion transport under high rate cycling conditions. Furthermore, major battery suppliers in China, Japan, and Korea have recently commenced the large scale production of LiFSI with their production capacity expanding^99^ Integrating these synchrotron characterization into this manufacturing step facilitates optimization of salt concentration and electrolyte formulations for industry-wide implementation.

*Ex situ* XPS is also used to investigate interphase products, providing substantial insight into the complex chemical species present. As shown in Figure 6e, Manthiram et al. investigated the CEI evolution on LiNi_0.7_Co_0.15_Mn_0.15_O_2_ electrodes subjected to the formation cycle and to 100 cycles.^197^ A native Li_2_CO_3_ film was identified on the surface of pristine particles. However, upon formation cycling, additional electrolyte decomposition products were observed, including LiF, MF_*x*_ with M = metal, Li_*x*_PO_*y*_F_*z*_, RCF_*z*_, and semicarbonates. Greater amounts of CEI reduction products are generated for cycled electrodes compared with after the formation process. Moreover, the thickness of the CEI is found to be particle-size-dependent; larger particle-size samples show fewer decomposition products based on the quantity of NiF_2_. This suggests that tuning particle size can critically influence CEI stability in terms of mitigating the degree of electrolyte decomposition. From a manufacturing perspective, particle size and morphology represent critical design parameters that must balance interfacial stability with transport kinetics. Increasing calcination temperature and sintering time generally promote grain growth and coarsening, leading to larger primary and secondary particles with reduced surface area.^210–212^ Based on the XPS observations, such particles tend to form thinner, more stable CEIs and exhibit less transition-metal dissolution, but the longer Li-diffusion pathways can penalize rate capability and fast-charging performance.^197^ Conversely, smaller or more highly faceted particles improve Li transport but exacerbate electrolyte decomposition because of their higher surface area. Thus, industrial cathode design requires optimizing both primary grain size and secondary particle architecture, along with accounting for the processing costs associated with calcination.

While *ex situ* UHV-based XPS is widely employed in battery research, it is inherently incompatible with volatile liquid electrolytes and is challenging to use for studying electrochemical interfaces under *operando* conditions. Synchrotron-based ambient-pressure XPS (APXPS) overcomes these limitations, enabling both *in situ* and *operando* characterization of electrode–electrolyte interfaces (Figure 6f.^213^). However, APXPS is still largely limited to electrolytes with low vapor pressure. As a result, most studies focus on propylene-carbonate-based or glyme-based electrolytes, restricting the ability to investigate widely used volatile carbonate systems.^127–129^

To overcome this limitation, *in situ* synchrotron X-ray reflectivity (XRR) is used to track SEI formation under more realistic conditions.^198,214^ XR enables quantitative analysis of SEI growth with subnanometer resolution. For instance, Cao et al. identified two SEI layers: one originating from electrolyte decomposition and the other from the native Si oxide layer, both resolved with subnanometer precision.^214^ This illustrates that removing the native oxide from Si anodes preferentially induces greater electrolyte decomposition, resulting in thicker and rougher SEIs. This can be a limiting factor during fast cycling. Conversely, Si anodes covered with thin native oxide form Li_*x*_SiO_*y*_ as a smooth SEI layer during activation, which is desirable for homogeneous (de)­lithiation. Importantly, this also eliminates the need for oxide removal with HF, as residual HF in industrial-grade electrolytes can degrade electrode surfaces.^215,216^ Despite its capabilities, X-ray reflectivity is suitable only for ideal systems such as large, flat Si wafers. These conditions deviate from those of practical composite electrodes composed of nanoscale particulates and porous architectures. Continued improvements in synchrotron optics and measurement geometries are expected to extend the applicability of XR to practical composite electrodes, where smaller probe sizes are required to accommodate heterogeneous microstructures.

The mechanistic understanding obtained from both *ex situ* and *operando* XPS/XR directly informs battery manufacturing. Formation cycling represents one of the most time-intensive steps in cell production, contributing up to 30% of fabrication cost consumption^17^ By pinpointing which interphase components are essential versus parasitic, these insights guide optimizations that shorten formation protocols and reduce failure rates. Furthermore, correlations between particle size, CEI/SEI chemistry, and long-term degradation provide design rules for high-Ni cathodes used in fast-charging EVs. Currently, quality control in the manufacturing process has relied mainly on EIS measurements and monitoring voltage/current responses as in-line quality control to correlate the observed impedance to the condition in the activation process.^3^ To accelerate the commercialization of more durable, cost-effective batteries at industrial scales, a comprehensive understanding of the products in the activation and cell conditioning process is highly needed through a combination of analytical techniques.^50^

### 4.4 Performance Evaluation and Manufacturing Feedback

Batteries operate under diverse and often extreme conditions, making performance evaluation critical for both fundamental understanding and practical applications. With the rapid growth in demand for high-power batteries, particularly in the EV market, it is highly desirable to understand battery-related phenomena occurring under high rates, elevated temperatures, and long-term cycling. Synchrotron-based characterization enables probing dynamic processes, identifying the origins of performance limitations, and guiding strategies through manufacturing feedback. The development of custom electrochemical cells has enabled reliable *operando* experiments, designed to preserve realistic or pseudorealistic electrochemical environments. Modified electrochemical cells^62^ and laminated pouch cells^217^ with X-ray-transparent windows serve as versatile platforms for real-time measurements using high-flux synchrotron beams. We discuss how findings from synchrotron-based techniques relate to manufacturing feedback and provide essential context for comprehensive performance evaluation.

#### 4.4.1 *In situ* and *Operando* Cell Design

While performance evaluation and *post-mortem* analysis can be performed *ex situ*, there are significant benefits to conducting *in situ* and *operando* analysis.^10^ This is because numerous artifacts can arise during sample preparation for *ex situ* analysis. Regions of interest may be lost during sample transfer and preparation. Samples may spontaneously self-discharge or undergo chemical changes, compromising their accurate representation of electrochemical states. This makes understanding behavior at different cycling stages challenging. Synchrotron beamtime is highly competitive, making extensive *ex situ* sampling across multiple states-of-charge (SOC) impractical. Nondestructive *in situ*/*operando* techniques are thus desirable to advanced understanding.

To conduct these experiments successfully, cell design becomes critical. Cell design should be developed in close consultation with beamline scientists because each beamline has different optics and energy ranges. Cells must incorporate a sufficiently wide X-ray-transparent region, or multiple regions, to permit both reflection and transmission geometries. For techniques requiring angular rotation, such as tomography, cylindrical cell geometries are preferred to maximize unobstructed angular access for 3D reconstruction. Accordingly, various cell types have been developed, including capillary cells,^218,219^ pouch cells,^61,217^ and the *in situ* X-ray AMPIX electrochemical cell.^220^

Heavy battery components such as copper current collectors and steel casings strongly attenuate and scatter X-rays and need to be considered in cell design. These materials strongly absorb X-rays and generate unwanted diffraction, reducing the quality of XAS, scattering, and imaging data. Cells typically require one or two X-ray-transparent windows, depending on whether reflection or transmission geometry is used. These windows are typically made of Kapton film, beryllium, or aluminum foil. In contrast, very high-energy X-rays can penetrate entire cells, expanding their applicability to more realistic formats.^221^ When constructing these cells, the windows should be impermeable to ambient air. Kapton films and beryllium are most suitable for these applications. However, beryllium is toxic and poses a safety hazard. Kapton films are soft, which makes it difficult to apply mechanical pressure during cell assembly.

Lastly, avoiding interferences from unnecessary cell components is important especially for the outgoing X-ray in transmission mode. This issue is particularly pronounced for cell layers containing heavy elements such as copper current collectors and stainless steel casings in cylindrical formats such as 18650, 21700, and 26650. Some techniques, including XAS, are not feasible in these configurations, and modified cells with X-ray-transparent windows are required for such experiments.^222^ Substituting Cu with Ti may be considered for detecting low-Z elements in battery components. In addition, in commercial cells, unless inactive components (PVDF and liquid electrolytes) are physically removed for beamline measurements, they generate diffuse background signals, and accurate subtraction is required to interpret X-ray data. From a manufacturing perspective, using cells “as-is” is ideal; however, not all advanced synchrotron techniques are compatible with fully sealed commercial cells. In practice, pouch-cell formats remain the most versatile platform due to their inherently X-ray-transparent configuration and compatibility with multiple *operando* geometries.^61,217^

#### 4.4.2 Microstructural Defects, Degradation Pathways, and Manufacturing Feedback

Recent manufacturing trends have driven the development of NMC with Ni contents exceeding 90% to reduce reliance on Co, a costly and supply limited element.^210^ Cobalt-free cathodes such as lithium iron phosphate (LFP) and lithium manganese oxide (LiMn_2_O_4_, LMO) with a spinel structure have also gained significant attention. As market demand shifts away from conventional NMC, a better understanding of electrochemical behavior and degradation mechanisms in emerging cathode materials is essential for achieving both high performance and cost efficiency. In addition, control over crystal structure and microstructure in cathode materials remains critical, including grain boundaries,^225^ oxygen defects,^226–228^ and transition-metal/Li disorder.^229^ Grain size and the number of grain boundaries present in primary cathode particles strongly influence crack formation and local strain evolution.^210^ Choices of raw materials, synthesis methods, and thermal treatments also determine defect density, grain boundary characteristics, and crystallographic texture,^58,210,230^ all of which impact mechanical properties and long-term stability. Thus, understanding how local strain and defect dislocation affect performance and how these defects and material properties are controlled by synthesis routes is essential for both manufacturing and research teams seeking to mitigate degradation in newly developed, cost-effective cathodes.

Advanced synchrotron techniques, including Bragg CDI, are indispensable for investigating these phenomena *in situ* and *operando*. They provide multiscale insights into structural evolution, defect formation, and material transport.^223^ They can, for example, reveal topological defects in battery electrodes.^65–67^ Through *operando* measurements, a single edge dislocation in LiNi_0.5_Mn_1.5_O_4_ cathodes was tracked within an individual nanoparticle at various charge states, and its dislocation field was used as a nanoprobe to determine the material’s local elastic properties.^65^ The results explain the material’s resistance to oxygen loss and structural collapse, and show that dislocations are stable at room temperature and mobile during charge and discharge, suggesting that defect motion can be a limiting factor in enhancing rate performance. Similarly, defects have been tracked in multiple cathode materials, including lithium-rich layered oxides.^66^ Defect formation pathways often originate from production choices such as precursor formulation and sintering conditions. For instance, twin boundaries and transition-metal/Li disorder introduced at different sintering temperatures are reported to be critical for structural stability in cathode materials.^57,58^ As a result, understanding the correlation between sintering conditions and the resulting defects is essential.^58,230^ Detailed cost evaluation of these processes and their associated material properties will also be critical for assessing manufacturing viability. These guidelines should be emphasized to enable industry to reliably scale high-Ni cathodes while retaining performance and safety.

One of the most prominent processing innovations inspired by these insights is the shift from polycrystalline secondary particles toward single-crystal cathode architectures.^231^ Conventional NMC synthesis produces agglomerated multigrain structures that accumulate intergranular cracking during high-voltage cycling.^210,232^ By contrast, single-crystal particles eliminate internal grain boundaries and therefore slow crack propagation and surface reconstruction.^233^ Achieving these morphologies, however, requires modified thermal-processing windows: higher calcination temperatures, carefully engineered dwell times, and flux-assisted environments (e.g., molten salt systems or LiO sublimation methods) to promote isolated crystal growth while suppressing secondary-particle fusion and impurity formation.^210,231,234,235^ These adjustments introduce cost and equipment challenges at scale, meaning that performance gains must be balanced with manufacturing efficiency, material utilization, and furnace longevity.

Synchrotron techniques, including XRD help evaluate the effectiveness of these process modifications by directly measuring lattice strain, defect density, phase purity in electrodes, and structural integrity under realistic cycling conditions.^10,82,236^ For instance, the observation of gliding in the operation of a single-crystal Ni-rich NMC cathode via multiscale synchrotron XRD techniques can provide guidelines for new cathode design.^237^ Huang et al. reported lattice distortion occurring in the cathode due to Li concentration gradients and were able to correlate the degradation mechanism. This provides potential solutions such as anion and cation doping, as well as the use of smaller active particles, to enhance structural integrity. However, these additional processes must also be evaluated in terms of their manufacturing cost. Enhanced structural integrity must offset the extra energy consumption, process complexity, and capital cost associated with thermal treatments.

Despite these advances, much of the existing synchrotron-guided synthesis research remains focused on laboratory or pilot-scale studies.^238^ Industrial-scale production introduces additional complexities: large coprecipitation reactors (for NMC synthesis), continuous furnaces with nonuniform temperature profiles, and broader precursor-quality distributions that generate defective or heterogeneous products.^99^ Even debris from battery components or equipment fragments may be introduced, affecting subsequent synthesis.^238^ Synchrotron studies have shown that subtle variations in calcination heating rates or precursor stoichiometry can yield secondary phases and compositional gradients that degrade performance.^231^ Similar considerations apply to other cathodes such as LiFePO, where *operando* synchrotron XRD has directly identified intermediate phases and reaction pathways during scale-up.^230^ These findings reinforce that manufacturing-relevant synchrotron studies are needed not only to engineer new materials but also to ensure quality, reproducibility, and safety as production volumes grow.

Not only material-level degradation but also cell-level degradation can be visualized, thus providing solutions for manufacturing. For instance, the excellent penetration capability of synchrotron high-energy XRD provides a powerful characterization tool that can guide manufacturing processes to mitigate battery aging effects and side reactions. Importantly, *in situ* and *operando* studies of lithium-ion batteries in various form factors are possible with high-energy XRD.^217,239,240^ In pouch cells comprising NMC532 cathodes and graphite anodes,^217^ small Li metal (110) reflections were identified in certain pixels of the 1D integrated diffraction patterns (Figure 7d), indicating the occurrence of Li deposition during fast charging alongside graphite intercalation reactions. Figure 7e further illustrates the heterogeneous distribution of lithium embedded within graphite and metallic lithium during cycling. The comparative mapping clearly demonstrates that lithium metal sites correlate with irreversible Li_*x*_C_6_ regions.

**Figure 7:**
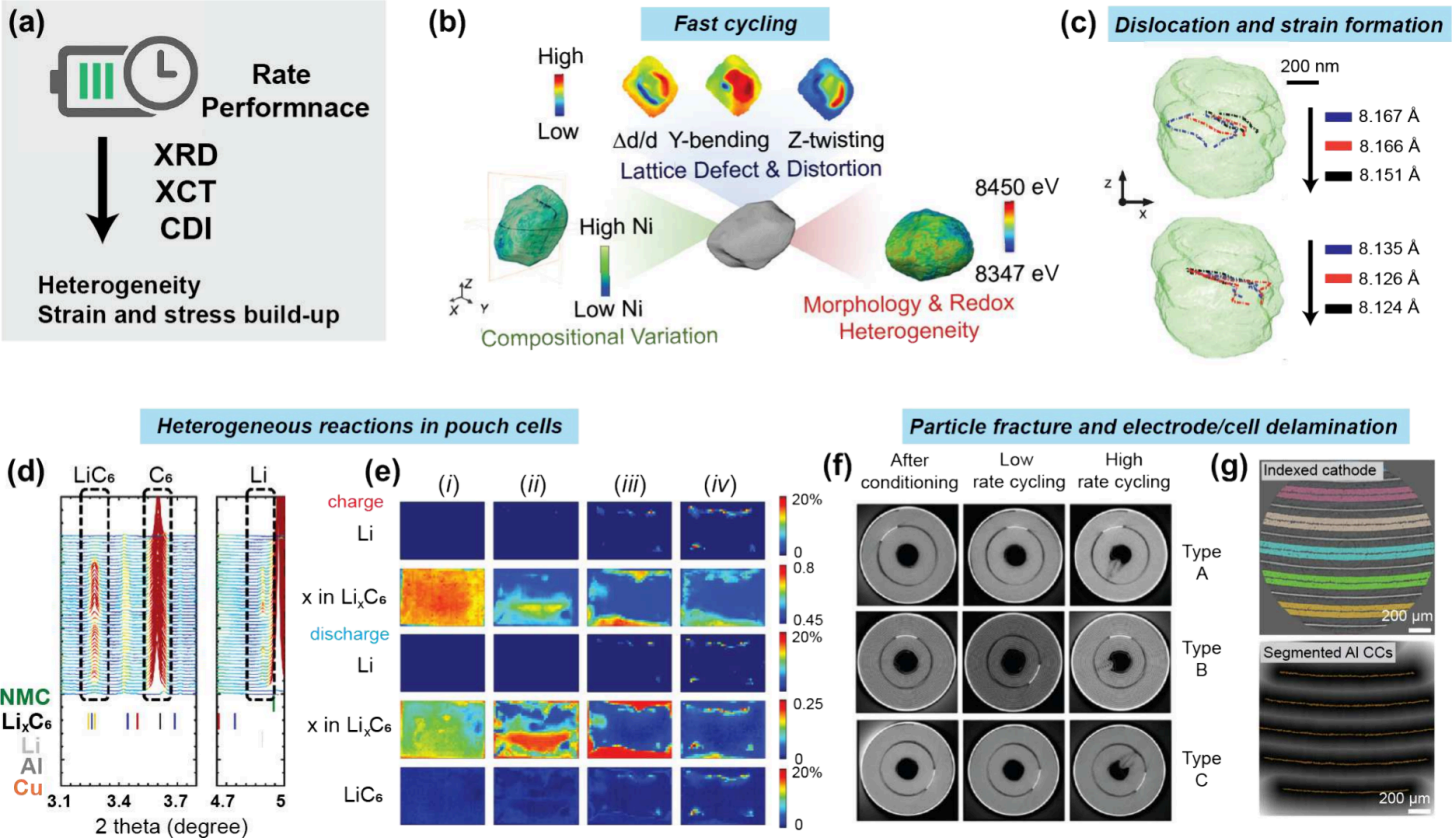
Rate performance analysis. (a) Schematics illustrating the key degradation mechanisms during high-rate cycling and the associated characterization techniques used to analyze them. (b) Reconstructed data from CDI contains information about lattice distortion of the lattice dimensions Δ*d*/*d* (strain), bending along the *Y*-direction perpendicular to the surface, the Z-twisting (in-plane), and the composition and morphology. (b) Reproduced with permission from ref 223. Copyright 2024 Elsevier, published under a Creative Commons license. (c) Defect (edge dislocation) location revealed by CDI (colored dots in the grain), at six different subsequent charge states (labeled with corresponding grain lattice dimension). The defect migrated toward the grain boundary. Adapted with permission from ref 65. Copyright 2015 the American Association for the Advancement of Science (AAAS). (d) Diffraction sets of a pouch cell from the LiNi_0.5_Mn_0.3_Co_0.2_O_2_ cathode, Li_*x*_C_6_ anode, Al, Cu current collectors. Formation of metallic lithium is attributed to the high overpotential induced by fast cycling. (e) Spatial maps showing Li deposition (row 1 and 3) and associated color maps of lithiated states of graphite (row 2 and 4) at different cycles: (i) 0, (ii) 3, (iii) 165, and (iv) 500. Residual lithium left in graphite (anode) indicating the irreversible lithium to cathode (row 5). (d,e) Adapted with permission from ref 217. Copyright 2021 American Chemical Society. (f) Radial CT scans of three differently aged 18650 cells (types A–C) taken at the center-height. Types A, B, and C use different cathode materials: Li_*x*_Mn_2_O_4_, Li_*x*_Ni_0.5_Mn_0.3_Co_0.2_O_2_, and Li_*x*_Ni_0.33_Mn_0.33_Co_0.33_O_2_/Li_*x*_Mn_2_O_4_ blend, respectively. The detail in the aging conditions can be found from the literature. Reproduced with permission from ref 59. Copyright 2014 Electrochemical Society. (g) Thickness tracking of double-coated cathode layers and its segmentation layers, which is revealed by X-ray tomography. Adapted with permission from ref 224. Copyright 2022 American Chemical Society.

In addition, quantification of capacity loss was enabled by *in situ* XRD in pouch cells under extremely fast cycling rates.^61^ Paul et al. suggested a methodology for tracking charge/discharge phenomena arising from SEI growth, lithium metal plating, trapped lithium in graphite. These observations from Paul et al.^61^ and Charalambous et al.^217^ carry significant implications for battery manufacturing and quality control. The ability to nondestructively detect localized Li plating and heterogeneous electrochemical reactions inside sealed cells enables early identification of electrode-design flaws, nonuniform current distribution, and mechanical misalignment in large-format batteries. Such insights can directly inform optimization of tab placement, electrode-stack tolerances, coating uniformity, and calendaring conditions to minimize local overpotential and mitigate plating-induced degradation. High-energy XRD has also been used to elucidate phase and lattice-parameter mapping through XRD-CT for commercial batteries such as AAA-type cells.^63^ However, widespread implementation remains challenging due to the highly absorbing steel casing, which produces intense unwanted scattering and complicates diffracted signal analysis. Furthermore, thick cell geometries introduce parallax artifacts, where scattered X-rays from the same 2θ arrive at different detector locations, distorting reconstructed diffraction patterns. To enhance diffraction signals, longer dwell times or larger voxel sizes are required, which limits both temporal and spatial resolution. As a result, most studies have been limited to single layer pouch cells (cathode/electrolyte/anode) and modified coin cells with Kapton windows. Advances in reconstruction algorithm is required to mitigate these issues.^63,241^

Furthermore, high-resolution and high-throughput micro-X-ray microscopy in various cell formats has also been developed to elucidate the degradation mechanisms during rate performance testing.^48,59,191,224,242^ Mechanical delamination and its associated failure mechanisms have been revealed with micro-XCT, as shown in Figure 7f.^59^ In this study, 18650 cells composed of different cathode materials (Li_*x*_Mn_2_O_4_, NMC532, and a NMC111/Li_*x*_Mn_2_O_4_ blend), labeled as types A–C, were aged under different cycling conditions ranging from low and high C-rate cycling. The CT images demonstrate that high-rate cycling induces the electrode buckling toward rotation axis, causing kinks in the electrodes and separators. In contrast, deformation is significantly less under low-rate cycling conditions. This observation illustrates that stress accumulation during cycling is more severe at high C-rates due to heterogeneous reactions in both the anode and cathode, ultimately resulting in partial delamination.^243^ Notably, such deformation is primarily observed in the inner region of the jelly roll in cylindrical cells, whereas pouch cells exhibit different degradation behaviors such as swelling, electrode bending, and uneven spacing.^48,242^ The use of a center pin was found to mitigate the structural collapse of cells during cycling.^59^ In addition, irreversible expansion of electrode layers under high C-rate conditions was also observed in 18650 cells.^224^ In this study, deep-learning segmentation of XCT images from commercial cells enabled quantitative tracking of distance changes in both anodes and cathodes across multiple cycles. Repeated imaging under aggressive charge–discharge conditions revealed electrode dilation and void formation (Figure 7g). Such void formation predominantly occurs near the copper current collector within the first few cycles, resulting in loss of contact between the active material and the conductive matrix. The localization of voids at the current-collector/electrode interface may be attributed to manufacturing-induced mechanical stress from electrode winding or to gas evolution during the formation process.^244,244^

#### 4.4.3 Thermal Stability and Runaway Analysis

One of the most pressing concerns in the development of battery technologies is the risk of thermal runaway, a chain reaction triggered by excessive heat, which poses serious hazards. In these systems, both battery performance and thermal stability directly influence overall cost, reliability, and safety.^6,246^ To address these issues, it is essential to gain a deeper understanding of the chemical reactions and structural transformations that occur within battery components at elevated temperatures. This has spurred a growing interest in advanced *in situ* and *operando* X-ray characterization techniques that can capture real-time changes in battery materials during operation. Among these, synchrotron XCT and XRD have emerged as a particularly powerful and versatile method in this application (Figure 8a).^7,247–249^ In this section, we aim to emphasize the significance of applying these techniques to analyze large-scale battery electrodes used in pouch cells, battery packs, and other commercially available formats.

**Figure 8:**
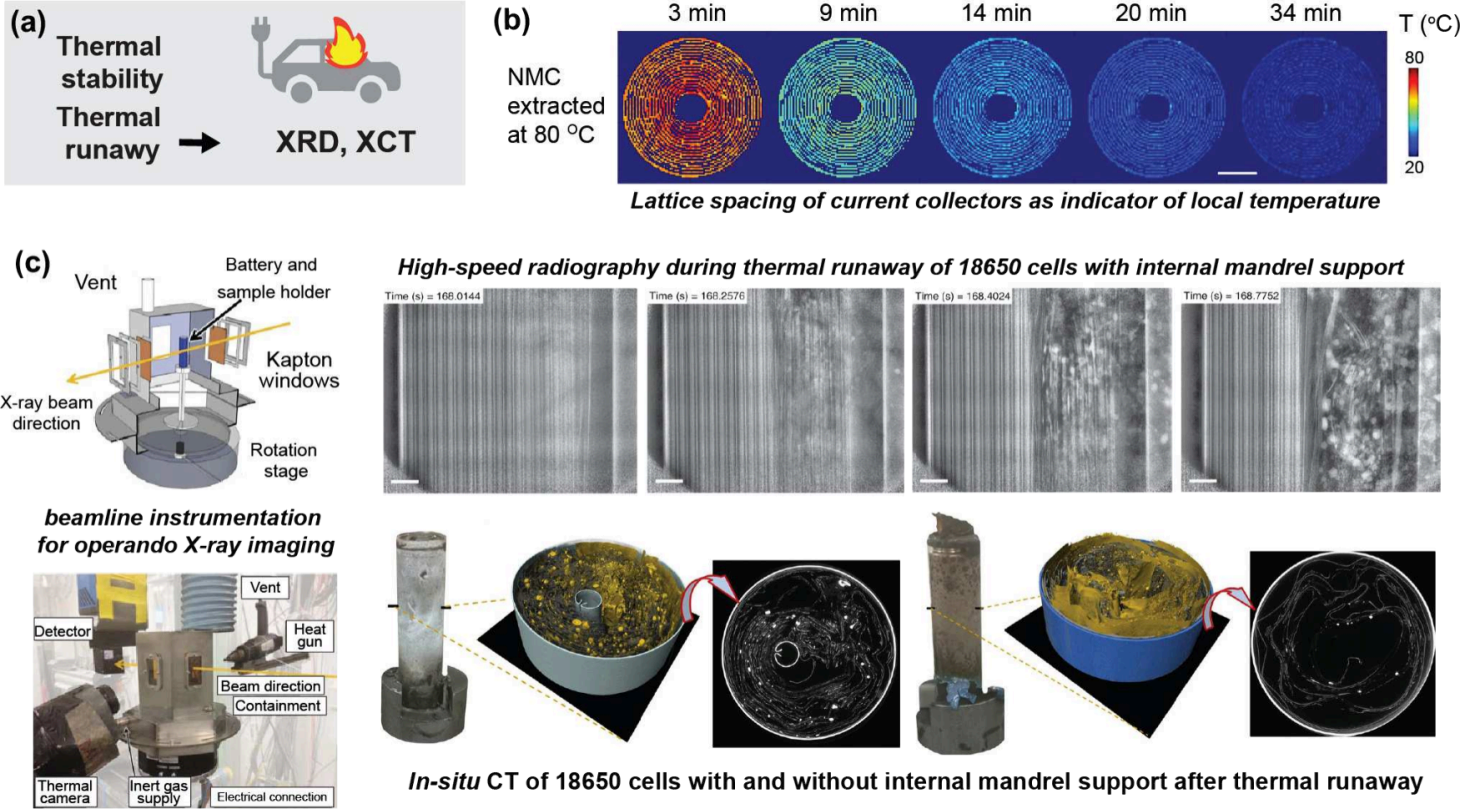
Thermal stability and runaway analysis through synchrotron characterization. (a) Schematic illustration of key synchrotron techniques used for analyzing thermal stability and runaway analysis in batteries. Analysis of thermal stability and thermal runaway of commercial battery materials and cells via (b) X-ray diffraction computed tomography. (b) Reproduced with permission from ref 13. Copyright 2023 Springer. (c) Arrangement of thermal runaway set-up for XCT experiments and its result. Reproduced with permission from ref 245. Copyright 2015 Springer.

Kong et al. addressed these risks from a manufacturing perspective, noting that standard safety testing, while required for certification, often fails to capture variability introduced during large-scale production.^7^ They highlighted that tab misalignment, coating irregularities, and separator defects, which are not consistently caught by batch testing, can lower the threshold for abuse-triggered failure, including thermal runaway. Characterization of every battery batch was recommended to establish baselines for acceptable thermal behavior, and CT was noted as one of the few tools capable of identifying these latent risks before they manifest during use.

CT has also been instrumental in distinguishing how different form factors respond to mechanical abuse at elevated temperatures. Lamb and Orendorff evaluated cylindrical and pouch cells under nail penetration and blunt impact, observing that solid-core constructions exhibited higher thermal stability due to reduced deformation under load.^249^ CT confirmed that failure onset was highly sensitive to internal geometry, with pouch cells requiring full-through penetration to initiate thermal events. These results suggest that mechanical robustness, rather than chemistry alone, plays a critical role in suppressing thermal runaway during physical stress or abuse. At the microscale, material heterogeneity has been shown to directly influence thermal gradients. Lu et al. reported that nonuniform pore structures within NMC particles led to localized ion flux heterogeneity under high-rate conditions, with narrow pores concentrating current and producing Ohmic heating.^191^ These hot zones increased the likelihood of electrolyte decomposition and were identified as potential initiation points for thermal runaway. The authors linked this behavior to fundamental design decisions in particle synthesis and electrode architecture, reinforcing the need to match porosity and tortuosity to the intended current load.

The temperature dependence of these failure modes has been further characterized through modeling grounded in CT-resolved geometries. Tranter et al. reconstructed spiral-wound 18650 cells from tomograms and applied a finite-difference resistor network model to simulate heat transfer and current flow under dynamic conditions.^250^ Their model predicted significant heterogeneity in local current density, especially in regions remote from tabs, where poor thermal connectivity amplified Joule heating. These predictions are consistent with experimental observations of asymmetric degradation and identify geometric layout, in addition to material selection, as a key contributor to runaway onset. Internal temperature gradients were shown to drive uneven stress and reaction kinetics, creating hotspots that exceeded safe operating thresholds under high current.

Despite the successes of CT for imaging battery architectures and buried microstructure, nonintrusively quantifying internal temperatures remains a formidable challenge. To address this, a recent study reported the first spatially resolved temperature maps of a commercial 18650 cell without disassembly or modification using X-ray diffraction computed tomography (XRD-CT), as shown in Figure 8b.^13^ By leveraging the thermally induced lattice expansion of metallic current collectors as a proxy for local temperature, the study produced accurate reconstructions of temperature fields inside intact commercial cells. This approach bypasses the need for embedded thermocouples, which disrupt the internal structure, and overcomes the limitations of surface probes that fail to resolve axial and radial gradients. The method demonstrated that heat accumulation during charging is strongly influenced by protocol, particularly under constant voltage conditions, and that degradation-driven increases in resistance amplify internal heating with cycle age. Crucially, the resulting temperature distributions were not uniform across the cell, showing clear spatial decoupling between heat generation and dissipation at high current. These findings establish XRD-CT as a nondestructive thermal diagnostic tool capable of identifying latent risks associated with localized overheating. Beyond measurement, the authors suggest this framework offers a path to validating thermal mitigation strategies in realistic formats, making it a valuable design and evaluation tool for commercial battery cells operating under high-rate conditions.

Beyond the influence of cell architecture on thermal runaway, XRD has been applied to reveal the underlying mechanisms and dynamics of active materials in response to thermal stimuli. Temperature-resolved XRD has been also applied to directly track structural stability of battery active materials under thermal abuse.^44,251^ By application of temperature-resolved XRD and residual gas analysis from coupled mass spectroscopy, the thermal stability, phase evolution, and oxygen release of charged LiNi_*x*_Mn_*y*_Co_1–*x*–*y*_O_2_ cathode materials was probed in detail by Bak et al. under controlled heating.^252^ Among the compositions studied, NMC811 exhibited the earliest onset of decomposition, with a sharp drop in the (003) reflection intensity beginning near 180 °C and a concurrent shift in lattice parameters consistent with oxygen release. This behavior, observed in the absence of electrolyte or external constraint, indicates that the delithiated Ni-rich layered oxide is thermodynamically unstable when heated and prone to structural collapse. The XRD methodology enabled identification of the temperature thresholds and the sequence of phase transitions unique to NMC811, establishing a benchmark for evaluating runaway precursors tied to material choice. As thermal excursions within cells are often initiated by localized overcharge or resistive heating, these data provide a material-specific reference for failure onset under abuse conditions.

Direct measurements of thermal response during abuse conditions have further confirmed the role of SEI breakdown and electrode–electrolyte reactions in initiating self-heating. A landmark study by Finegan et al. combined high-speed synchrotron CT and thermal imaging to follow thermal runaway in *operando*, revealing structural evolution with subsecond resolution (Figure 8c).^245^ The authors compared two commercial 18650 cells, one with a central mandrel and one without, and identified profound differences in deformation and venting behavior. In the supported cell, the internal architecture remained largely intact up to and through venting, with localized gas generation leading to controlled release through the pressure relief vent. Structural collapse was minimal, and the exothermic reactions were allowed to run to completion within the casing. By contrast, the unsupported cell underwent severe internal deformation, with gas-induced delamination and collapse of the spiral-wound layers. This structural failure disrupted current collector alignment, accelerated short circuit formation, and prematurely terminated reaction propagation. These findings demonstrate that mechanical design features, such as a central mandrel, can significantly alter the trajectory of thermal failure by maintaining geometric coherence under pressure.

Together, these studies show that thermal runaway is rarely the result of a single failure point. Instead, it arises from the coupling of material, geometric, and electrical asymmetries, many of which develop slowly and accumulate with use. Synchrotron analyses, at both the active material and cell level, provides the resolution and context needed to identify where these instabilities begin, how they evolve, and under what conditions they cross critical thresholds. As battery production scales and new chemistries with ultrahigh energy density are pursued, detecting and mitigating risks of thermal runaway during the manufacturing process will become crucial for minimizing risk across diverse application environments. Integrating X-ray-based inspection with thermal modeling offers a pathway toward early stage qualification and long-term safety assurance.

## 5 Outlook: Future Perspective of Synchrotron Experiments for Battery Manufacturing

### 5.1 High-Throughput Synchrotron Characterization for Battery Manufacturing

Traditionally, synchrotron beamlines were optimized for low-throughput, user-operated experiments, but the expanding needs of the battery industry now demand higher levels of automation, throughput, and standardization. High-throughput characterization has become more commonplace at synchrotrons through the use of robotics,^255^ mail-in experiments, standardized cells,^220,256^ and increasingly sophisticated data-streaming and real-time analysis.^257^ Many of these advances were pioneered by the macromolecular crystallography community^258^ and its interactions with the pharmaceutical industry.^259^ Early experiments incorporated the use of robots to automate sample transfer and alignment,^260^ which quickly evolved into mail-in programs that now support the majority of macromolecular users across synchrotrons worldwide.^258,261^ Similar concepts have expanded to high-resolution powder diffraction,^262^ surface scattering and spectroscopy,^263^ computed tomography,^264^ and spectroscopy.^265^ These developments form a natural foundation for manufacturing-oriented workflows, where consistency, repeatability, and statistical sampling are critical for quality control at production scale.

In parallel, high-throughput electrochemical characterization has been explored for combinatorial battery materials^266,267^ and battery cells, as illustrated in Figure 9a.^268,269^ In these setups, tens or hundreds of samples are measured simultaneously with varying electrode/electrolyte compositions or electrochemical boundary conditions. These types of combinatorial experiments are well-suited for the rapid data acquisition possible at synchrotron light sources. Moreover, high energy techniques, such as powder diffraction or white beam micro-CT, are amenable to standard cell formats used by industry that could be adapted to mail-in studies. The bottom panel in Figure 9b embodies this approach, where synchrotron powder X-ray diffraction (PXRD) through a simple 2032-format coin cell can be measured in <10 s. As shown in the top panel of Figure 9b, expanding this cell format to a rack of 96 channels would require only 10–15 min to measure the entire set, which is well within the time frame of a typical *operando* charge–discharge cycle. The ability to characterize such a large number of cells in parallel could accommodate multiple users simultaneously. These types of large data sets could also provide training data for autonomous experiments or correlating electrochemical and structural features important for lifecycle prediction.^270^ This level of parallelization directly reflects industrial bottlenecks, where thousands of cells per day require rapid quality control, and could support multiuser or multiformulation screening in a production-relevant manner. This setup can be further expanded to commercial cells such as cylindrical-type and pouch cells, enabling high-throughput characterization that is more amenable to industrial use.

**Figure 9:**
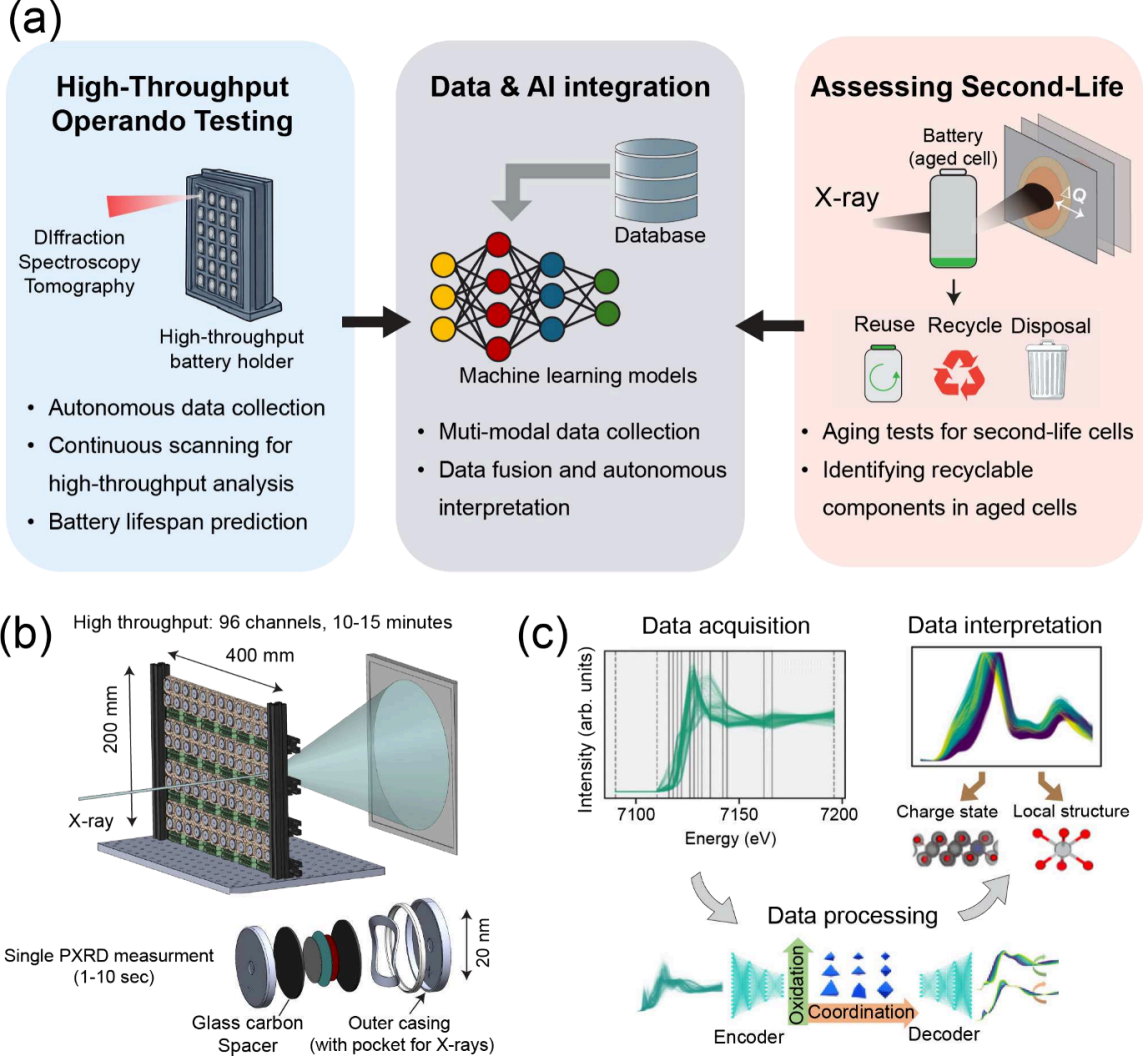
(a) Schematic overview of future perspective of synchrotron enabled workflow for battery manufacturing. (b) Using high energy synchrotron X-rays (>50 keV), standard coin cell formats can be used for powder X-ray diffraction (PXRD), which only takes seconds to measure. For this cell, the inner spacers are replaced by glass carbon disks to provide uniform stack compression, like previous *in situ* cell designs. Taking advantage of the fast measurement time, an array of 96 cells could be used to enable higher throughput. (c) Diagram illustrating the key applications of machine learning methodologies for synchrotron-based characterization. Reproduced with permission from ref 253. Copyright 2024 Royal Society of Chemistry, under CC BY-NC 3.0. The bottom panel was reproduced with permission from ref 254. Copyright 2023 American Physical Society.

### 5.2 Machine Learning-Enhanced Synchrotron Characterization

The rapid increase in data volume and complexity in synchrotron-based measurements presents major challenges for battery-manufacturing applications, where decisions must be made rapidly and reproducibly. When applied to electrode production, slurry mixing, drying, or calendaring, synchrotron data contain rich information about particle distribution, binder network evolution, phase transformations, and defect formation. Traditional analysis pipelines rely on manual interpretation, which severely limits throughput and introduces variability. As high-throughput and *operando* measurements produce multidimensional data sets (spectra, diffraction, tomography, scattering, multimodal combinations), maintaining accuracy and consistency across diverse chemistries and production conditions becomes increasingly difficult. Therefore, to enable closer integration between synchrotron facilities and battery manufacturers, experimental workflows must become more accessible, streamlined, and automated, lowering the expertise barrier for routine use.^271^

Machine learning (ML) offers a powerful route to accelerate and standardize synchrotron workflows directly relevant to manufacturing, as shown in Figure 9c. ML models can be trained on large experimental or simulated data sets (e.g., XASdb^272,273^ ) to automatically identify material properties,^274^ correlate features across different data modalities,^275,276^ and extract hidden trends that may not be apparent through manual investigation. Importantly, these models can generalize across new electrodes or processing conditions with minimal human supervision, providing a consistent QC framework across production batches. Furthermore, representation learning techniques such as autoencoders can extract latent features from complex spectra, enabling property-spectrum mapping,^254^ clustering^277^ and anomaly detection across large sample pool. By leveraging ML, researchers can accelerate the analysis pipeline, improve reproducibility, and unveil deeper scientific insights from high-throughput or large-scale synchrotron experiments, all of which are critical to the process optimization in high throughput production levels.

In data acquisition, ML enables adaptive and efficient experimental control. Subsampling methods like recursive feature elimination can reduce acquisition time by selecting a minimal, informative subset of XAS energy points while preserving interpretability, reducing acquisition time by up to 80%.^253^ Anomaly detection models, such as deep convolutional neural networks trained on raw diffraction images, can identify anomalies from abnormal beam output or data collection with 90% accuracy.^278^ By integrating ML into the beamline data acquisition tools, acquisition parameters can be adjusted in real time to improve signal-to-noise ratio, minimize redundancy, and reduce beam damage. These strategies support closed-loop experimental systems that increase throughput, minimize manual intervention, and optimize data quality.

For data processing, ML facilitates automated, high-fidelity extraction of structural and chemical information. Segmentation algorithms based on convolutional neural networks are effective at isolating regions of interest in tomography and microscopy data so that detailed microstructural analysis is enabled. For example, 3D U-Net architectures combined with watershed segmentation have been used to achieve accurate grain-wise segmentation in time-resolved CT data, even when trained on limited ground truth from 3D XRD.^279^ Denoising models further enhance imaging and spectroscopic data by suppressing noise and artifacts with minimal manual correction.^280^ ML-driven feature extraction also supports the direct identification of oxidation states^274,281,282^ and local structural distortions.^274,281,283^ Coherent X-ray scattering (CXS) techniques such as CDI and XPCS produce speckle patterns that encode the full electron density information, with dynamic information for XPCS. AI/ML is used in CXS to solve the phase retrieval problem,^284^ as well as to directly extract dynamics using physics-informed neural networks.^285^ Together, these techniques improve reconstruction fidelity and make large-scale data processing scalable and reproducible.

Multimodal measurements combining techniques such as XRD, XAS, XPS, XPCS, and CDI have been more common to probe structural, and dynamic properties of battery electrodes *operando* in a complementary fashion. Despite the availability of these diverse data streams, most ML applications for interpretation are designed using single-modal inputs, targeting tasks such as oxidation state prediction^254^ or structural changes.^286^ Pioneering work by Na Narong et al.^276^ shows that multimodal ML pipelines can guide experimental design by revealing which modalities contain the most informative features that can be used for manufacturing-relevant predictions. Future approaches will benefit from cross-modal attention mechanisms and unified representations that integrate spatial, spectral, and temporal information. This mirrors the complexity of real manufacturing environments where multiple quality control tools feed for decision-making process.

The real power of ML-driven synchrotron lies in the accelerated feedback to manufacturing. By correlating *in situ* structural and chemical characterizations with cell performances, the integration of ML can directly optimize electrode formulation, drying, and calendaring parameters, enabling fast quality control and process optimization on the production line. In this way, what begins as high-throughput characterization at a beamline becomes a seamless loop back into scalable battery production, accelerating both discovery and deployment. Looking ahead, close collaboration between industry and academia will be essential for the development and deployment of scalable ML tools in large-scale testing environments for battery manufacturing. Such efforts will accelerate data-driven experimentation and ultimately lead to autonomous experimental platforms.

### 5.3 Second-Life Applications

The lifespan of LIBs in EVs is typically about 10 years, after which the remaining achievable capacity is around 70% of the initial value. Owing to the rapidly growing number of EVs in use, a significant volume of retired battery packs is expected in the near future. As batteries reach the end of their first life, many still retain considerable usable capacity, motivating strong interest in second-life deployment for grid storage and other low-C-rate applications.^287^ From a manufacturing perspective, second-life pathways offer clear economic and environmental benefits by reducing material waste and lowering demand for critical raw materials. However, determining which cells remain suitable for repurposing requires a precise understanding of internal degradation mechanisms. Several degradation models have been proposed for evaluating batteries for second-life applications, each emphasizing different pathways such as loss of lithium inventory in anode or cathode particles, electrolyte depletion, and other chemical or mechanical aging processes. Data-driven approaches have also explored degradation assessment through parametrization and neural-network-based prediction.^288,289^ However, conventional electrochemical measurements derived from these models lack spatial resolution and therefore cannot reliably capture localized failure modes.

Synchrotron-based nondestructive techniques, including X-ray diffraction computed tomography, microfocused X-ray diffraction, and X-ray absorption tomography, now provide a unique pathway to reveal the hidden structural and chemical changes that dictate residual value. To enable meaningful correlations between cycling history, synchrotron-based characterization, and predictive modeling, operational history and cell specifications must be systematically cataloged. Such comprehensive metadata allow researchers to link degradation signatures to specific use conditions, thereby improving both mechanistic understanding and the reliability of second-life prediction models^287^

One of the earliest demonstrations of the potential for nondestructive insight came from a study^290^ (the third panel in Figure 9a). Using synchrotron X-ray diffraction and three- dimensional tomography, the formation of lithium hydroxide on lithium metal anodes was investigated in a lithium oxygen battery. Contrary to expectations, the passivation layer did not fully isolate the electrode. Instead, it formed a porous network that allowed ions to continue migrating through narrow channels of LiOH, reaching to metallic lithium.^291^ For second life qualification, this implies that certain transformed regions may still be operational depending on their spatial structure, expanding the criteria for reuse. Later *operando* studies on lithium oxygen batteries have further shown that degradation is a spatially dynamic process.^292^ Using depth-resolved synchrotron diffraction, researchers visualized how lithium peroxide nucleates uniformly in early cycles and later accumulates in dense regions and insoluble carbonate tends to accumulate in the separator region and eventually plugs the ion transport between the electrodes. These deposits, largely invisible to bulk techniques, severely restricted ion transport and increased cell resistance. It produces additional small crystallites that eventually clog pore channels. This spatial phase evolution, reported in Shui et.al,^292^ indicates that passivation is not an all-or-nothing event but rather the outcome of local growth dynamics and electrode architecture. It emphasizes the need for whole-cell diagnostics to evaluate all components that can influence long-term performance. In the context of second life evaluation, these findings show that separators and interfaces may harbor critical bottlenecks that limit reuse even when electrodes appear functional.

The repair of costly active materials represent another promising pathway for improving the economic viability of second-life and recycling strategies.^57,293^ Upon arrival at a recycling facility, batteries are typically disassembled, and valuable components, including active materials, are selectively extracted. For example, LFP can be recovered by removing the polymer binder followed by pyrolysis and alkaline treatment to eliminate residual carbon and current-collector materials.^287^ These extracted active materials can then be repaired through reintroduction of transition metals and lithium. Various approaches have been demonstrated, including high-temperature solid-state methods^294^ and molten-salt-assisted regeneration techniques.^295^ While these materials are restored, their material properties including grain size, texture, defects change from the initial states depending on the processing conditions.^57,293^ Synchrotron techniques can play an important role in supporting LIB recycling by enabling direct visualization of structural recovery, quantification of defect evolution, and validation of regeneration pathways under realistic processing conditions.

Taken together, these studies demonstrate the power of synchrotron-based nondestructive characterization in evaluating electrode integrity across full battery systems. By reconstructing internal lithium distribution, identifying phase accumulation, and capturing spatial asymmetries, these techniques offer an unparalleled view of degradation mechanisms. They move diagnosis beyond single value metrics and toward detailed structural maps that inform decision making in second life logistics. Rather than merely detecting failure, these methods expose the history and geometry of degradation. This level of insight is essential for building reliable reuse pipelines and accelerating the transition to circular battery economies.

### 5.4 Radiation Damage and Reliability

The demand for user-friendly synchrotron facilities is increasing, driven by the need for high-throughput and automated measurements as well as analysis assisted by machine-learning. As more data are collected and processed by users with limited expertise, particularly under increasingly realistic and complex experimental conditions, ensuring data reliability becomes more challenging. This issue is especially critical for battery materials, which are highly sensitive to radiolysis and can be altered by excessive accumulated X-ray dose. For example, LiF has been long proposed as a stable SEI component in LIBs, but was recently shown to be, in some cases, an artifact arising from beam-induced decomposition and ion sputtering of lithium salts.^296^ High-resolution techniques such as nanotomography often expose samples to extremely high doses, which can burn the material or modify its chemical structure.^101^ Even relatively low-resolution techniques, such as conventional XAS, can induce measurable chemical changes during prolonged irradiation.^297^ These observations highlight that many experimentally observed features may be influenced by X-ray exposure itself, and that electrochemical reactions monitored under *operando* conditions may be dose-dependent.

Despite this, users are often tempted to use high photon flux to achieve high spatial or temporal resolution. Dose-dependent artifacts have been reported in *operando* studies of NMC, LFP, and carbon electrodes, where excessive irradiation can delay key reactions.^298–300^ In some cases, it can suppress electrochemical activity, or even induce artificial phase transformations. Relatively lower doses can still modify peak widths and intensities, producing apparent heterogeneity that may not reflect intrinsic material behavior.^299,300^ Recent studies have also emphasized the importance of dose rate, not only total dose, in governing beam-induced effects.^299^ As a result, it is recommended that total radiation dose be monitored for each measurement or electrochemical cycle, and that experiments be designed to keep cumulative exposure below material-specific thresholds to ensure accurate interpretation of battery chemistries. Strong collaboration with beamline scientists should be emphasized, even though some industrial users may have concerns about sharing proprietary details.

The reliability of synchrotron-based characterization is not limited by beam dose. The conditions required for many advanced techniques are often far from those encountered in manufacturing. For instance, conventional XPS cannot operate under ambient pressure or in the presence of volatile electrolytes,^124^ and although APXPS partially alleviates these constraints, the resulting measurements still deviate from true cell environments.^127^ Consequently, SEI information extracted from XPS may not fully represent the chemistries that form under practical battery operating conditions, even though the technique remains uniquely powerful for mechanistic SEI studies. Similarly, single-layer pouch cells are frequently used for X-ray scattering and imaging experiments because of their reduced attenuation and simplified geometry,^61^ whereas wound and multilayer commercial pouches dominate industrial production. These discrepancies highlight that insights gained from highly controlled experiments cannot always be directly transferred to roll-to-roll environments, where pressure, temperature gradients, and mechanical deformation evolve on different length and time scales.

Unlike academia and national laboratories, industrial teams often operate under proprietary constraints and may not openly share detailed cycling histories, failure modes, or processing recipes. Thus, improving representativeness requires closer coordination between synchrotron facilities and industrial partners, not simply for broader data dissemination. Encouragingly, detailed descriptions of battery cycling conditions, radiation dose, and sample chemistries are increasingly being formalized into shared, interoperable data structures. Large community initiatives, such as the BIG-MAP program within the European battery research ecosystem, provide frameworks for data exchange, and collaborative protocol development across institutions.^79,301^

While synchrotron-based techniques offer unparalleled insights on the operation principles of batteries, many demonstrations to date rely on simplified cell architectures or modified formats, and translation to commercially manufactured cells remains an active area of development. Furthermore, there is a growing concern on whether the resultant characterization outputs are reliable or not.^79^ These challenges highlight the need for community discussion and coordinated research efforts aimed at unifying or standardizing experimental cell designs, such that results obtained at different facilities or using different techniques can be directly compared and artifacts arising from cell geometry or construction can be minimized. Such practices could ultimately help guide the development of manufacturing strategies in industry, which fosters collaboration between industry and academia

## 6 Conclusion

Integration of synchrotron X-ray characterization into the development and manufacturing of batteries has become indispensable. By providing multiscale insights into chemical, structural, and morphological structure across the entire manufacturing process, these techniques enable the detection of latent cell failure modes and the optimization of process parameters under realistic conditions. The advent of new generation synchrotron sources with high brilliance and high coherence has expanded *operando* diagnostic tools for performance evaluation. Future efforts should prioritize close collaboration between industry and academia to develop standardized workflow for sample designs and X-ray experimentation. The application of machine learning for real-time data analysis will further enhance the utility and the impact of synchrotron X-ray characterization in advancing battery manufacturing.
